# Using Free-Living Heart Rate Data as an Objective Method to Assess Physical Activity: A Scoping Review and Recommendations by the INTERLIVE-Network Targeting Consumer Wearables

**DOI:** 10.1007/s40279-024-02159-1

**Published:** 2025-02-02

**Authors:** Moritz Schumann, Joshua F. Feuerbacher, Lars Heinrich, Marcos Olvera-Rojas, Alessandro Sclafani, Jan Christian Brønd, Anders Grøntved, Brian Caulfield, Ulf Ekelund, Wilhelm Bloch, Sulin Cheng, Luis B. Sardinha, Francisco B. Ortega

**Affiliations:** 1 Department of Molecular and Cellular Sports Medicine, German Sport University, Cologne, Germany; 2 Department of Sports Medicine and Exercise Therapy, Chemnitz University of Technology, Chemnitz, Germany; 3 Department of Physical Education and Sports, Faculty of Sport Sciences, Sport and Health University Research Institute (iMUDS), University of Granada, Granada, Spain; 4 Department of Sports Science and Clinical Biomechanics, University of Southern Denmark, Odense C, Denmark; 5 Insight Centre for Data Analytics, University College Dublin, Dublin, Ireland; 6 Department of Sport Medicine, Norwegian School of Sport Sciences, Oslo, Norway; 7 Department of Chronic Diseases, Norwegian Institute of Public Health, Oslo, Norway; 8 Faculty of Sport and Health Sciences, University of Jyväskylä, Jyväskylä, Finland; 9 Exercise, Health and Technology Centre, Department of Physical Education, Shanghai, Jiao Tong University, Shanghai, China; 10 Exercise and Health Laboratory, CIPER, Faculdade de Motricidade Humana, Universidade de Lisboa, Lisbon, Portugal; 11 CIBER de Fisiopatología de La Obesidad y Nutrición (CIBEROBN), Instituto de Salud Carlos III, Granada, Spain

## Abstract

Wearable technologies open up new avenues for the assessment of individual physical activity behaviour. Particularly, free-living heart rate (HR) data assessed by optical sensors are becoming widely available. However, while an abundancy of scientific information and guidance exists for the processing of raw acceleration data, no universal recommendations for the utilization of continuous HR recordings during free-living conditions are available. Towards Intelligent Health and Well-Being: Network of Physical Activity Assessment (INTERLIVE^®^) is a joint European initiative of six universities and one industrial partner. The consortium was founded in 2019 and strives towards developing best-practice recommendations in the context of consumer wearables and smartphones. The aim of this scoping review (following PRISMA-ScR procedures) and recommendations was to provide best-practice protocols for deriving individual physical activity profiles from continuous HR recordings by wearables. The recommendations were developed through an initial scoping review, grey literature searches of promotional material and user manuals of leading wearable manufacturers as well as evidence-informed discussions among the members of the INTERLIVE^®^-network. The scoping review was performed on the generic domains required for physical activity assessment, namely: (1) ‘assessment of maximal heart rate’, (2) ‘determination of basal and/or resting heart rate’ and (3) ‘heart rate-derived intensity zones’, for which we finally included a total of 72, 2 and 11 eligible papers, respectively. Gathering recent knowledge, we provide a decision tree and detailed recommendations for the analysis of free-living HR data to derive individual physical activity profiles. Moreover, we also provide examples of HR-metric calculations that help to illustrate data processing and reporting.

**Supplementary Information:**

The online version contains supplementary material available at 10.1007/s40279-024-02159-1.

## Key Points

**Table:** 

Free-living heart rate (HR) data assessed by optical sensors are becoming widely available but guidance on the use to assess free-living physical activity (PA) on the basis of continuous HR is lacking.
Utilizing free-living HR to assess individual PA patterns requires also standardized procedures for the measurement/estimation of maximal HR, basal/nocturnal HR and HR-based intensity zones.
Combining the knowledge retrieved from systematic literature searches and discussions within the INTERLIVE network, this paper provides a decision tree and detailed recommendations for the analysis of free-living HR data to derive individual PA profiles.

## Introduction

The development of wearable technology is evolving rapidly [1, 2], providing an easy access to extensive data related to physical activity, fitness, sports performance and health. Wearable devices often comprise of inertial sensors [i.e. inertial measurement units (IMU)] for the estimation of steps by means of acceleration and/or optical sensors [i.e. photoplethysmography (PPG)] that allow for estimations of heart rate (HR) through readings of the pulse wave [3]. In contrast to a 12-lead electrocardiogram (ECG) that directly traces ventricular depolarisation, PPG is based on the absorption and reflection of emitted light by the blood, where the transmitted or reflected light is modulated by the systolic variations in blood volume that, in turn, is closely linked to HR [3]. Owing to improved battery life and gradual reductions in size and weight [2], wearables are especially advantageous for continuous recordings of bodily functions during free-living conditions over multiple days or even weeks.

Most commonly, collected PPG data are processed through algorithms that are rarely publicly disclosed. Moreover, data quality and validity may differ considerably between devices and are commonly unknown to the user [4]. As such, feedback provided for end-users is typically on the basis of summary data, such as average HR, but it remains unknown how many data points are underlying these data (e.g. non-wear time or periods with low data quality/missing data) [4–7]. While it has been shown that summary data may be sufficient to improve adherence to regular physical activity and may even lead to improved health-related outcomes, such as body composition [8], these data may not be sufficient for clinical purposes or research settings. This is especially the case when individual activity patterns have to be assessed, which is why for research purposes, mostly devices that provide raw acceleration data have been used [9–11].

While an abundancy of scientific information and guidance is available for the processing of raw acceleration data [9–12], it has to be acknowledged that devices providing high-quality data are expensive and may, thus, not be preferred for health promotion purposes. Additionally, other weaknesses of acceleration data regarding the wearing position [9, 11, 13], as well as the detection of low-acceleration type of activities (e.g., cycling, resistance training or uphill versus downhill running) have previously been identified [9]. These constraints somewhat limit the generic use of accelerometers and inertial measurement units, supporting the use of other data assessable by wearable devices. In this context, the use of continuous HR to quantify physical activity patterns during free-living (i.e. time spent at different intensities per day) may be promising. However, to the best of our knowledge, currently no guidance for the standardized intensity classification of free-living HR data exists.

Previous research discussing exercise intensity thresholds has mainly focused on the intensity distribution of individual exercise sessions in an athletic context [14–16]. As a result, several methods were proposed, that are based on important metrics, such as maximal HR (HR_max_), maximal oxygen consumption (VO_2max_) or the HR and VO_2_ reserve (i.e. HR_max_ – resting HR and VO_2max_ – resting VO_2_, respectively). For example, the American College of Sports Medicine (ACSM) suggested the classification of very light, light, moderate, vigorous and near to maximal/maximal exercise intensities (Table 1) [17]. However, these models are commonly based on assumed disturbances of the physiological homeostasis and their validity is still a matter of debate [15]. Such an approach would at a minimum require cardiopulmonary exercise testing including breathing gas analysis or the assessment of blood lactate concentrations, both of which are labour intensive. Moreover, free-living physical activity is often characterized by an abundance of light activity which may result in an overrepresentation of the light intensity zone [18], and thus not necessarily providing a sufficient resolution to differentiate between individual physical activity profiles.

**Table 1:** Examples of exercise heart rate (HR)-zones suggested by different authors. NB: these zones are provided for illustration purposes only and are not necessarily endorsed by the INTERLIVE^®^-network

ACSM [9]	ACSM [9]	Roete et al. [41]	Sylta et al. [40]	Jamnick et al. [7]	Rønnestad et al. [39]
%HRR	%HR_max_	%HR_max_	%HR_max_	%HR_max_	%HR_max_
< 30 (very light)	< 57 (very light)	50–59	55–72	65–75 (recovery)	60–82
30–39 (light)	57–63 (light)	60–69	72–82	75–80 (extensive)	
40–59 (moderate)	64–76 (moderate)	70–79	82–87	80–85 (intensive)	83–87
60–89 (vigorous)	77–95 (vigorous)	80–89	87–92	85–92 (training)	88–100
≥ 90 (near max.)	≥ 96 (near max.)	90–100	92–97	> 92 (interval training)	

Considering the lack of guidance on the use of free-living HR metrics, this paper aims to provide evidence-informed recommendations for the profiling of free-living physical activity patterns on the basis of HR. Further elaborating on the above, this also includes the development of standardized approaches for the assessment or estimation of maximal as well as basal and/or resting HR. The provided guidelines are targeted at researchers and manufacturers, as well as sport and clinical practitioners and aim to facilitate a standardized and harmonized approach for obtaining HR-derived activity profiles assessed by wearables.

## Background: The Interlive^®^ Network

INTERLIVE^®^ is a joint initiative of the University of Lisbon (Portugal), the German Sport University (Germany), University of Southern Denmark (Denmark), Norwegian School of Sport Sciences (Norway), University College Dublin (Ireland), University of Granada (Spain) and Huawei Technologies Finland. The consortium was founded in 2019 and combines expertise in sports and exercise medicine, health epidemiology, health technology and biostatistics. The main aim of the consortium is to develop best-practice protocols for evaluating the validity of consumer-grade wearables as well as to provide guidance on the utilization of wearable-derived data to foster a widespread use of physical activity indicators. To date, INTERLIVE^®^ has published recommendations for determining the validity of consumer-grade wearable devices for HR [4] and step counts [5] as well as more indirectly derived metrics, such as energy expenditure [7] and maximal oxygen consumption (VO_2max_) [6].

## Methodological Approach

In an initial network-meeting held on 24 October 2022, the process for the development of recommendations for the objective profiling of physical activity on the basis of free-living HR data was discussed. In this meeting, an iterative three-step process for the development of recommendations was agreed upon, consisting of a (1) a scoping review with systematic literature search, (2) a grey literature search of user manuals and other promotional materials of leading wearable manufacturers and (3) evidence-based discussions among the INTERLIVE^®^-network. On the basis of the a priori knowledge of each consortium member, the consortium also agreed on three domains to be targeted during the scoping review that were deemed relevant for assessing free-living physical activity on the basis of HR: (1) methods to assess or predict HR_max_, (2) methods to assess or predict resting and/or basal HR and (3) methods to determine HR-zones.

The scoping review with systematic literature search was conducted by a sub-group of the INTERLIVE^®^ network (M.S., J.F.F. and L.H.). Only papers proposing or validating methods to determine, predict or estimate HR_max_ (i.e. domain 1), basal or resting HR (i.e. domain 2) or HR intensity zones (i.e. domain 3) were included. The systematic literature search was conducted on 6 December 2022 and updated on 26 September 2024, using the checklist for Preferred Reporting Items of Systematic Reviews and Meta-Analysis Protocols extension for Scoping Reviews (PRISMA-ScR). The PubMed/MEDLINE, ISI Web of Science, and SPORTDiscus databases were searched for the previously identified three domains. The search strings were specifically adapted to the search requirements of each database (Online supplementary data, Table S1). Additionally, reference lists of included studies were screened for potentially missing papers. A flowchart of the search process and study selection for the three domains is shown in online supplementary data (Figures S1a to S1c). Papers were eligible when their full text was available, papers were listed in one of the searched databases and were written in English language. No limit in terms of the publication date was in place.

All results from the online search were saved, imported and further analysed using the Rayyan tool for systematic reviews. The literature search process was performed independently by two authors and included removing duplicates and screening titles, abstracts and full texts. Potential conflicts were resolved by consulting with a third author. Article characteristics such as authors, title, type of paper (e.g. original study, systematic review, narrative review) and main results (e.g. formula for predicting maximal heart rate, proposed heart rate-based intensity zones) were extracted separately for each domain.

In parallel with the scoping review, grey literature searches were also performed by another sub-group of the INTERLIVE^®^-network (M.O.R., A.C. and F.B.O.). This search specifically targeted user manuals, technical documentation and other promotional material of established manufacturers. In this process, we summarized grey literature information for an entry-level as well as medium and high-grade model of selected manufacturers that held large market shares from 2020 to 2022 [19] and have comprehensive manuals and technical documentation of their products publicly available. Namely these manufacturers were Amazfit, Apple, Fitbit, Garmin, Huawei, Polar, Samsung, Suunto and Xiaomi. Devices from the recent product range of each manufacturer were classified into ‘entry-level’ as well as ‘medium and high-grade’ models on the basis of their pricing. The cheapest device in the current product range was categorized as ‘entry-level’, the most expensive as ‘high-grade’, and the device with a price closest to the midpoint between these two as the ‘medium-grade’ model. The extracted data from grey literature included information on the options to extract free-living continuous data (i.e. irrespective of HR that is measured during specific exercise sessions) as well as possible predictions of maximal and/or resting/basal HR. Since continuous HR data are typically displayed in mobile applications, we expanded this grey literature search to the following prominent fitness apps: Zepp (Amazfit and Xiaomi), watchOS 9 (Apple), Fitbit App, Garmin Connect, Huawei Health, Polar Flow, Samsung Health and Suunto App.

The results of the scoping review and grey literature search were then discussed with the entire consortium in another online meeting held on 21 April 2023. In this meeting, a first draft of recommendations for the objective profiling of physical activity by HR was established, which was further refined by selected members of the network (M.S., J.F.F., L.H. and F.B.O.) and subsequently shared for revisions with the entire consortium.

## Current State of Knowledge

### Results of the Scoping Review with Systematic Literature Search

We identified a total of 72, 2 and 11 eligible papers for the domains ‘HR_max_’, ‘basal/resting HR’ and ‘HR-zones’, respectively.

*Identified Papers for the HR*_*max*_ *Domain*

Of the 72 reviewed papers for the HR_max_ domain, 47 attempted to derive or propose unique HR_max_ prediction equations or models (online supplementary data, Tables S2a to S2c), while 25 papers solely aimed at evaluating the validity of already existing equations in different populations (online supplementary data, Table S3). Out of the 47 papers, a total of 106 unique HR_max_ prediction equations were extracted. Of these, 63 equations target healthy non-athletic populations (extracted from 31 papers), 28 equations target athletic populations (extracted from 9 papers), and 15 target diseased populations (extracted from 11 papers). Note that five papers provided equations for multiple populations [20–23].

*Identified Papers for the Basal and Resting HR Domain*

In contrast to HR_max_, only limited direct evidence exists on the methods to assess basal and resting HR in children [24] as well as young men [16, 24]. Logan et al. [24], derived resting HR by measuring HR in the morning within 30 min of awakening and compared this to variations in the lowest HR assessed through continuous recordings throughout an entire school day [24]. Depending on the method used, a variance between the morning and day measures of up to 35% was observed. In a similar manner, Davis and Convertino [16] compared nocturnal HR with HR determined in one of the following four conditions: (1) directly after awakening using palpation, (2) after 15 min of rest in a supine position, (3) in a seated position and (4) after 10 min of standing in a quiet room. The lowest HR was observed during the night but did not statistically differ from the resting HR assessed by palpation immediately after awakening. Both the night and morning condition differed substantially from all other conditions.

*Identified Papers for the HR-Zones Domain*

Additionally, only few papers were identified that directly addressed the determination of HR-zones to cluster exercise or physical activity on the basis of their intensity. The majority of included papers focused on HR-zones that were assessed in accordance with physiological variables, such as %VO_2max_ [16], measures of the ventilatory/lactate thresholds [25–29], or the point of metabolic acidosis [29, 30]. In addition, studies have compared a percentage range above resting HR compared with a percentage of HR_max_ in cardiac patients [31] or used the HR at the critical power to demarcate heavy from severe exercise intensities in young women [32]. Furthermore, another study compared HR-based indices to global positioning system (GPS)-derived training load in professional soccer players [33]. Finally, we identified two review papers that focused on the applicability of different exercise prescription methods based on HR [15, 34]. The ACSM provides recommendations for the classification of exercise intensity on the basis of HR_max_, VO_2max_ and heart rate reserve (HRR) [34]. Jamnick et al. concluded that estimating training intensity zones based on maximal anchors, such as a percentage of HR_max_ or VO_2max_, is inaccurate because this method is not consistent with physiological parameters that delineate these intensity zones [15]. However, this is typically discussed in well-trained athletes and whether this holds true for untrained or sedentary populations remains unknown.

Taken together, the findings of our scoping review indicate an abundancy of scientific data on HR_max_ assessment, which may be used to directly derive recommendations. Conversely, the scientific evidence on the recommended methods of assessing resting and/or basal HR appears to be insufficient to draw conclusions. Similarly, only few papers have directly addressed the determination of HR-zones. The majority of these papers clearly outline the physiological challenges associated with the definition of intensity thresholds. Moreover, in all included papers, HR-zones were primarily developed to quantify exercise intensity rather than free-living physical activity.

### Results of the Grey Literature Search Focusing on Information Provided by Manufacturers

A summary of the data retrieved from user manuals of established manufacturers is provided in online supplementary data Table S4a. While 10 models did not specify which equation is used to predict the HR_max_, the remaining 17 models appear to use the Fox et al. [35] prediction equation ‘220 – age’. This is of particular concern as the lack of scientific basis for this equation was previously shown [36] and numerous population-specific and validated alternatives are available (online supplementary data Tables S2a to S2c). Additionally, some manufacturers, such as Polar, allow users to input the individually assessed HR_max_ manually.

Of the 27 reviewed wearables, only 4 models specified their methodology to derive resting HR. All three included models by Polar require the user to lie supine and breathe calmly for 3–5 min. Additionally, the high grade Amazfit model uses the nocturnal HR measured over at least 5 h to estimate the resting HR. All other models simply instruct the user to wear the device continuously throughout the day without further specification.

Concerning the analysis of continuous HR data, Amazfit, Apple, Fitbit, Samsung and Xiaomi allow for a user-friendly download of continuous data through the corresponding app (online supplementary data Table S4b). Huawei and Garmin allow for an export of these data through an additional developer tool, while Polar and Suunto currently only provide an option to download HR data from individual training sessions. Interestingly, out of the nine manufacturers searched, only five (Amazfit, Fitbit, Garmin, Polar and Xiaomi) [37–39] currently display free-living activity zones on the basis of continuous HR, with considerable inconsistencies among the classifications used (Table 2).

**Table 2:** Examples of free-living HR-zones currently used by leading manufacturers. NB: these zones are provided for illustration purposes only and are not necessarily endorsed by the INTERLIVE^®^-network

Fitbit	Zepp (Xiaomi and Amazfit)	Garmin	Polar
%HRR	%HR_max_	HR and ACC based*	HR and ACC based*
40–59 (fat burn)	> 50 (relaxed)	Below training	Resting
	50–60 (light)	Warm up	Sitting
60–84 (cardio)	60–70 (intensive)	Easy	Low
	80–90 (aerobic)	Aerobic	Medium
> 85 (peak)	90–99 (anaerobic)	Threshold	High
	100 (maximum)	Maximum	

*Manufacturers use a combination of HR and accelerometer (ACC) data to determine daily physical activity zones; exact HR zones are not specified

### Special Considerations for Physical Activity Profiling

On the basis of the scoping review with systematic literature search, grey literature search and a priori knowledge of the INTERLIVE^®^-network, the following considerations concerning the three domains (i.e. ‘HR_max_’, ‘basal/resting HR’ and ‘HR-zones’) provided the foundations for the developed recommendations of profiling free-living physical activity on the basis of continuous HR measures. An additional overview on variables that require attention is provided in Table 3.

**Table 3:** Factors affecting maximal and basal/resting heart rate

Maximal heart rate		Basal/resting heart rate	
Stable	Transient	Stable	Transient
Age ~ [52, 54, 112, 119–123] Sex ~ [23, 47, 50–52, 54, 55, 58, 66, 79, 112, 124–126] Day-to-Day variability↓↑ [48] Individual preconditions [17] Body mass [55] Body fat [22, 55] Ethnicity ~ [50, 51, 55, 67, 127–130] Training status ~ [23, 47] Disease status: Cardiovascular↓[35, 54, 59, 60, 65, 68–70, 73] Chronic fatigue syndrome↓[131, 132] Obesity↓[22] Cerebral palsy↓[72] Mental retardation↓[20] Smoking status ~ [58, 133] Chronic medication↓↑^a^ [17]	Increment duration ~ [15, 40–43] Stage duration ~ [15, 40–43] Overall test duration ~ [15, 40, 42] Exercise mode: Treadmill↑[44, 45, 52] Cycle ergometer↓[17, 44–46, 52] Sport specific↑[47] Insufficient recovery & fatigue↑[17, 43] Nutritional status [17] Environmental conditions [17] Activated muscle mass [47, 52, 60, 66, 134, 135] Activity type↓↑[47, 52, 60, 66] Transient medication↓↑^b^ [17]	Age [24, 84, 86, 136] Sex [87, 137] Day-to-day variability [138] Time of day [16] Cardiovascular fitness [139] Body composition [140] Environmental conditions [95] Training status [104, 141] Chronic medication↓↑[17]	Acute infections and disease↓↑[142–144] Psychological stress↑[145] Environmental conditions [146] Ambient light [147] Sleep quality [92] Sleep phase [92] Deviations from regular bedtime [148] Body position: [85, 149–151] Supine↓ Sitting↑ Physical activity: [95, 96, 152] Time since Rest period Intensity Duration Food intake↑[153–155] Nicotine↑[156, 157] Caffeine ~ [158–161] Transient medication↓↑[17]

↑ Increase, ↓ decrease, ↓↑ increase or decrease depending on specific conditions, ~ inconclusive evidence

^a^Medication that is taken continuously over a longer period of time to treat chronic disease

^b^Medication that is taken only once or over a short period of time to treat acute disease

NB: Appendix A (Table A.1) of the ACSM’s ‘Guidelines for Exercise Testing and Prescription’ [17] provides an overview of common medications and their expected (chronic) influence on the HR kinetics

*HR*_*max*_ *Assessment*

The majority of studies reporting HR_max_ rely on HR values obtained from a common incremental VO_2max_ test, such as the Bruce protocol [17]. However, it remains inconclusive whether HR_max_ is affected by the protocol characteristics. In two studies, no differences were observed between the HR_max_ obtained with 1 and 3-min increments [40, 41]. However, Machado et al. [42] reported an optimum increment duration of 2 min. Furthermore, it was shown that HR obtained in traditional incremental tests may be lower (5.76 ± 2.81 bpm) than that obtained from specifically designed 3–4 min all-out performance [43].

Since the optimal protocol for assessing HR_max_ remains unknown, it is reasonable to suggest that HR_max_ should be assessed through a graded maximal exercise test that is in line with the population specific standard exercise testing procedures recommended by the ACSM [17]. It should also be considered that longer increments may lead to premature fatigue and prevent the attainment of HR_max_ [15, 40, 42]. Therefore, shorter stage durations (≤ 2 min) or ramp protocols (with 30–60 s per increment) are preferred. Similar to VO_2max_ testing, a total duration of 8–12 min seems optimal to assure maximal cardiovascular exertion without premature fatigue [15]. Irrespective of the protocol used, secondary criteria to determine maximal voluntary exhaustion may be applied. These commonly include respiratory exchange ratio (RER), subjective ratings of perceived exertion or blood lactate concentrations [15, 17].

In addition to the protocol characteristics, the selection of the exercise mode seems crucial. As HR is dependent on muscle mass involvement [44, 45], treadmill tests are considered a gold-standard. VO_2max_ values achieved using treadmill protocols tend to be up to 20% higher compared with cycling protocols [45]. Importantly, a HR_max_ derived from a treadmill protocol likely best reflects the HR during free-living conditions for most populations and testing e.g. on a cycle ergometer may cause early local muscle fatigue, preventing cardiorespiratory exertion [17]. Cycle ergometers may, however, be considered an alternative for clinical populations that are unable to perform treadmill protocols [17, 46]. Furthermore, for specifically trained athletes a sport specific ergometer may be used to attain the actual HR_max_ [47].

Irrespective of the testing mode, standardisation of the testing conditions is required. For example, fatigue and insufficient recovery from previous exercise may acutely affect the HR response. In this context, Ingjer [43] found that after one or two days of high intensity training only few individuals were able to reach their previously tested HR_max_. Therefore, no strenuous exercise should be performed a minimum of 24 h before the testing procedure [17]. Detailed recommendations for other factors, such as nutritional status, environmental conditions and individual preconditions, are found elsewhere [17]. Importantly, once the test is performed in standardised conditions, the mean day-to-day-variability of HR_max_ in healthy but untrained populations appears to be as low as 1% [48].

Maximal tests are often labour-intensive and may require extensive equipment. Moreover, without medical clearance these are typically restricted to young and healthy populations [17]. Thus, prediction models were developed to estimate HR_max_ on the basis of individual characteristics, such as age and sex. Our scoping review revealed that nearly all (*n* = 44) papers that derived prediction equations included age among other variables, while 42 papers used age alone (online supplementary data Table S2a–c). However, even though univariate age-based equations are most frequently used because they are simply applicable and relatively easy to understand, the majority of these equations seem to be prone to large prediction errors [36]. Thus, several studies aimed to develop multivariate (*n* = 13) equations or population-specific univariate equations to increase the predictive capacity by including additional factors, comprising of sex [23, 47, 49–54], resting HR [55–60], HR during submaximal tests [57, 61–65] and type of activity [47, 52, 60, 66]. Additionally, individual studies also considered HR variability [62], body mass and body fat [55], ethnicity/nationality [50, 67], specific diseases [20, 59, 65, 68–72] and medication [73]. As it has been shown that the most common prediction equations are valid for adults but not for children and adolescents [72, 74–80], separate equations for children and adolescents have been proposed, including variables like resting HR, maturity offset, body mass and body fat as additional variables [55, 56, 78].

*Basal/Resting HR Assessment*

Our scoping review revealed a dramatic lack of scientific guidance on how to assess resting and/or basal HR. Generally, the lowest levels of HR refer to a condition where the metabolic requirements are minimal [81]. Thus, it appears reasonable to take advantage of methods that are used to assess basal and/or resting metabolic rate. Basal metabolic rate was initially defined as the minimal rate of energy expenditure compatible with life [82]. This is typically assessed as the heat production (or VO_2_ as the surrogate of energy expenditure) at rest and in a supine position in strictly controlled laboratory environments, including a fasted state with controlled environmental conditions [83]. Basal metabolic rate, in turn, needs to be distinguished from nocturnal metabolism, which typically appears to be lower [83], also indicating that the lowest values of HR are expected to be observed during sleep. In fact, it was shown that the HR assessed by palpation directly after awakening was statistically lower than HR measured during a later time of day [16], indicating that lowest values of HR were obtained nocturnally.

Importantly, basal and nocturnal metabolic rate differ from resting metabolic rate, which is typically assessed in less strict laboratory conditions, i.e. at any given state of rest throughout the day. As such, resting HR appears to be higher than nocturnal or basal HR [84]. Nonetheless, resting HR is considered a key vital sign and is a well-established predictor of all-cause and cardiovascular mortality [85] and commonly included in various studies [85]. It appears that resting HR assessments generally involve a resting period between 5 and 10 min [86–88], while the participants are in a supine position [89–91] and the HR is obtained from the last minute of the measurement.

When assessing nocturnal HR, the effects of sleep quality need to be considered. It has been shown that deep and quiet sleep [i.e. non-rapid eye movement (NREM)] is associated with a lower HR compared with restless and superficial sleep [i.e. rapid eye movement (REM)] [92]. In healthy individuals, good sleep quality includes about four to six cycles per night, with each cycle lasting an average of 90 min [93]. However, it remains unknown how many sleep cycles are required for overnight HR assessments. There are a number of studies that incorporated measures of nocturnal HR variability, with a typical duration of 4 h commencing 30 min after reported bedtime [94–99]. This seems reasonable considering the length of individual sleep phases (i.e. 90 min) and is also in line with data showing that the early phases of sleep appear to be a quiet sleep period [100].

*HR-Zone Determination*

With the rapid increase of commercially available wearables that allow for continuous free-living HR assessment, individual physical activity profiles may be determined. In athletic populations, HR-zones are often aligned with changes in the metabolism, represented by ventilatory and/or lactate thresholds. Indeed, the majority of studies identified by our scoping review used indices of the metabolism as a determinant of HR-zones [15, 16, 25–28, 30, 33]. Especially in endurance athletes, typically a three-zone model is used to cluster the exercise intensity into light (below the first ventilatory/lactate threshold), moderate (between the first and second ventilatory/lactate thresholds) and severe/vigorous (above the second ventilatory/lactate threshold) [14]. However, while these submaximal anchors were used to describe exercise intensity by numerous studies, the actual validity is still debated [15]. The criticism brought up refers to whether these zones make a demarcation that reflects actual homeostatic disturbances [15]. In fact, numerous methods for the determination of lactate thresholds exist [15] and, thus, errors in the definition of the lactate thresholds can have a detrimental effect on the classification of intensity distributions. Moreover, graded exercise testing to voluntary exhaustion is needed to accurately determine these thresholds [101, 102], thus limiting its utilization to specific populations.

For example, our grey literature search of leading manufacturers revealed various fixed HR zones that are on the basis of percentages of the HR_max_ or HRR and are used for scientific purposes (Table 1) or provided to end-users (Tables 2, 4). However, the underlying assumptions for deriving these zones remain unknown, leading to inconsistencies among different methods. This also further underlines that the suggested zones do not reflect the underlying metabolic fluctuations. That is, large ranges of fixed percentages of HR_max_ were previously associated with both the ventilatory threshold (60–90% of HR_max_) and the maximal lactate steady state (75–97% HR_max_) [103]. Thus, it appears that fixed percentages of HR_max_ to prescribe exercise intensity do not demarcate distinct physiological characteristics [30].

**Table 4:** Examples of exercise HR-zones currently used by leading manufacturers. NB: these zones are provided for illustration purposes only and are not necessarily endorsed by the INTERLIVE^®-^network

Fitbit	Zepp (Xiaomi and Amazfit)	Garmin	Polar
%HRR	%HR_max_	%HR_max_	%HR_max_
40–59 (fat burn)	> 50 (relaxed)		
	50–60 (light)	50–60	50–60 (very light)
60–84 (cardio)	60–70 (intensive)	60–70	60–70 (light)
	80–90 (aerobic)	70–80	70–80 (moderate)
> 85 (peak)	90–99 (anaerobic)	80–90	80–90 (hard)
	100 (maximum)	90–100	90–100 (maximum)

In addition to the methodological difficulties outlined above, exercise intensity is also affected by resting HR which, in turn, is dependent on individual factors such as the training status [104]. Consequently, for the same HR_max_, individuals with a lower resting HR (i.e. trained individuals) will be able to produce more work compared to individuals with a higher resting HR (i.e. untrained individuals). Thus, simply quantifying exercise intensity by percentage of HR_max_ will easily lead to an over- or underestimation of the actual activity performed. To overcome these issues, often the HRR has been used to quantify physical activity in various populations [28, 34]. In fact, guidelines for the quantification of physical activity on the basis of HRR are also provided by the ACSM, suggesting five zones as follows: (1) near maximal to maximal (≥ 90% HRR), (2) vigorous (60–89% HRR), (3) moderate (40–59% HRR), (4) light (30–39% HRR) and (5) very light (< 30% HRR) [17]. However, in an earlier position stand even the ACSM clearly outlines the limitations that are associated with the classification of these zones, also when based on HRR [34]. This is because large inter- and intraindividual differences exist in the relationship between HR_max_ and VO_2max_ as well as HRR and VO_2reserve_ (i.e. VO_2max_ − VO_2rest_) [41, 105, 106].

Nevertheless, it appears that the use of HRR seems most appropriate for the purpose of assessing exercise or activity intensity. However, the intensity-zones used so far mainly serve to distinguish high-intensity activities/exercise from moderate- and low-intensity activities/exercise that are expected to lead to an improvement in cardiorespiratory fitness [34]. However, free-living physical activity is characterised by an abundance of low-intensity activity below the first threshold [18]. It is, therefore, questionable whether there is sufficient resolution of these activities on the basis of established intensity-zones. In fact, it is important to bear in mind that even lower intensities in the range of 30–45%VO_2reserve_ can lead to an increase in cardiorespiratory fitness [107] that may not be adequately represented by commonly suggested HRR zones, especially in untrained or diseased populations. Accordingly, a higher resolution of the low-intensity activities is required to accurately describe free-living physical activity. Moreover, particular settings that outline individual changes in daily activity patterns, rather than describing the intensity of a single exercise session, may benefit from a more detailed depiction of these activities.

Considering the demands on methods to quantify free-living physical activity by continuous HR on the one hand, while bearing in mind the physiological variability on the other hand, it appears that the selection of the preferred method highly depends on its purpose. For example, applying the ACSM guidelines also allows to assess adherence to common physical activity guidelines, such as e.g. outlined by the World Health Organization (WHO) [108]. For healthy adults, although caution should be exercised as these recommendations are based on self-reported data, 150 min of weekly moderate activity are suggested, where moderate is defined as an energy expenditure of 3–6 metabolic equivalents (METs). According to the ACSM intensity thresholds, this would be reflective of 40–59% HRR [17]. However, if the aim is to compare physical activity patterns inter- and/or intra-individually, dividing HRR into clusters of 10% (i.e. 10, 20, 30% etc.) might be an appropriate approach to display individual activity profiles. This approach would allow for a higher resolution of individual free-living activity patterns by using smaller increments that also allow the capture of small changes in activity intensities. More importantly, this approach does not rely on universal group-based thresholds. In fact, utilizing relatively fine increments for the quantification of continuous HR would also be in line with the subjective rates of perceived exertion, as assessed by the modified rating of perceived exertion (RPE) scale (Borg CR10) [18, 109, 110]. The universal use of the RPE scale, however, is debated, as large inter-individual variability exists, especially among individuals with different fitness levels [111]. Nevertheless, aligning the 10% clusters derived from the HRR with the ten increments of the RPE scale may aid in interpreting the individual activity profiles.

## Evidence-Informed Recommendations

**Fig. 1:**
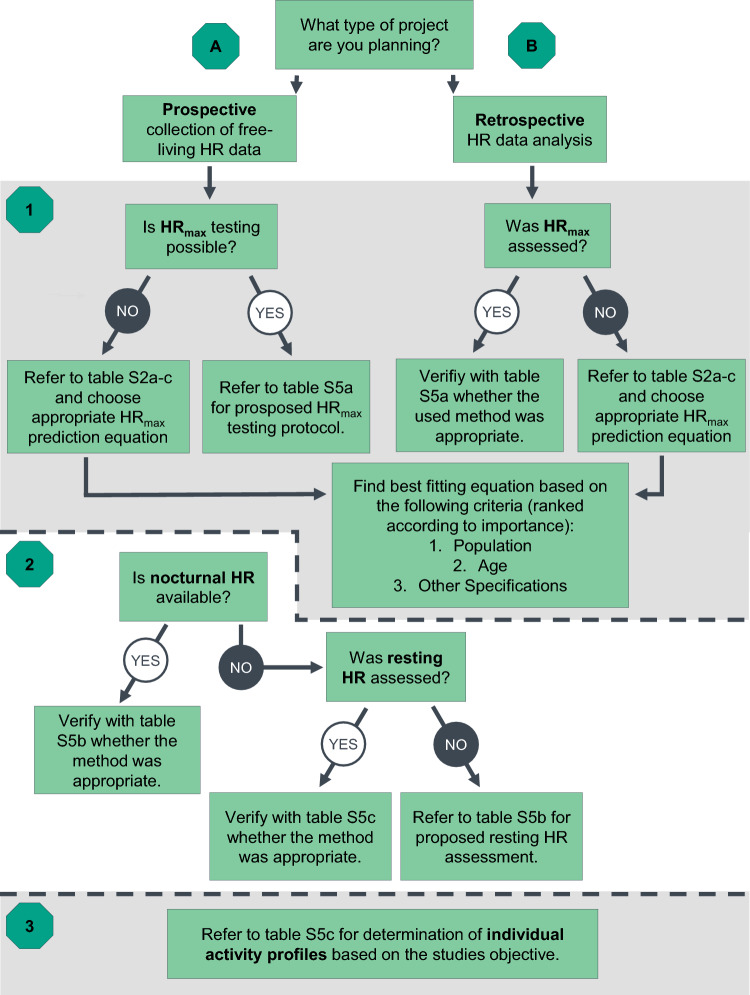
Decision tree for the profiling of free-living physical activity by continuous heart rate (HR) measures on the basis of the calculation of heart rate reserve (HRR). Branch A illustrates the necessary steps that should be planned in prospective data collections. Branch B provides guidance for the analysis of already existing data sets (i.e. retrospective analysis). To select the appropriate equation to estimate HR_max_, a three-step process is suggested: (1) population (i.e. ‘healthy’, ‘athletes’ or ‘diseased’), (2) age, (3) other specifications, such as further characteristics of the athletic background (i.e. type of sport) or disease (i.e. type of disease)

In Fig. 1, we present a decision tree that illustrates the necessary steps to derive physical activity profiles from free-living HR in prospective data collections or existing data sets. Detailed recommendations on each of these levels are provided in Tables 5, 6 and 7. Importantly, our recommendations may only be applied if the pre-processed continuous HR data (as opposed to daily summary data) can be accessed. Thus, caution is warranted when selecting appropriate devices for the free-living HR assessment. This also includes gathering possible information on the accuracy of the selected device [4–7]. It should be also noted that the handling of continuous HR data requires special attention. However, providing standards for the mathematical and statistical processing, such as data extraction, smoothing and outlier detection was beyond the scope of the present paper.

**Table 5:** Proposed protocol for the assessment of maximal heart rate (HR_max_)

Methodological domains	Methodological variables	Protocol considerations	Reporting considerations
Experimental HR_max_ assessment	Pre-test preparation	Depending on the population of interest, a medical screening is recommended. The Guidelines for Exercise Testing and Prescription of the American College of Sports Medicine provide an overview [17] Participants using regular medication that affects cardiovascular function (e.g. beta blockers) should be asked to continue intake as usual If medically possible, avoid transient medications (e.g. NSAIDs, stimulants, antihistamines, antibiotics, cold medication) for at least 24 h before testing or longer if necessary owing to the half-life of the drug Participants should refrain from intense physical activity 48 h prior to the maximal test Participants should be informed about the testing procedures and preparations. A sufficient amount of sleep (e.g. 7–9 h for adults [162]) prior to the scheduled testing Restrict nutritional intake 3 h prior to the test to avoid gastric complications. Habitual caffeine intake is of no concern	Pre-test standardisation should be reported
	Test standardisation	Testing should be performed in standardised ambient conditions (temperature 20–22 °C, humidity < 60%) We recommend performing the test at a time the individual is habituated with and well-nourished Use a 12-lead ECG or chest strap that has been shown to have an excellent agreement with a gold standard to obtain HR. An overview on suitable devices is presented elsewhere [4] As long as a validated ECG or chest strap is used for the assessment of HR_max_, sampling rate is of no concern	Report ambient conditions, time of day and the device used for HR recording (including the sampling rate and version of the firmware)
	Maximal test design	An 8–12-min graded exercise test to exhaustion should be performed (duration of each increment ≤ 2 min) Generally, a treadmill test should be preferred. For clinical populations unable to walk or run, a cycle-ergometer test is acceptable. For specifically trained athletic populations, a sport specific ergometer and/or protocol should be preferred Allow for a population-specific warm-up (2–5 min) at self-selected low pace Verify voluntary exhaustion and other maximal criteria according to recommendations provided by the American College of Sports Medicine [17]	Report specifics of the testing protocol (i.e. step length, overall test duration and velocity or power per increment) Report voluntary exhaustion criteria
	Data analysis	Select the highest HR value provided NB: in cases where data are provided beat-by-beat, conversion to HR data for every second is required. Data should be smoothed by 15–30 s rolling averages. The highest 15–30-s rolling average should be considered as HR_max_ [6] If data are obtained by a PPG-based wearable device, it is advised to choose a device that uses a constant high sampling rate. However, in any case, information on the sampling rate has to be obtained prior to selecting a device. Data points that are within the reported sampling rate range should be normalized to the highest sampling rate on the basis of the previous recorded HR value. Missing data (i.e. defined as deviations from a given sampling) should be excluded from the analysis	Report software/application version for data processing (including download and analysis) Report HR_max_ and how it was calculated (e.g. highest 30 s rolling average)
Prediction of HR_max_	Selection of prediction model	The selection should be based on the target population Please refer to supplementary online data, Tables S2a–c for an overview of existing models. For cross-validation of existing models, check supplementary online data, Table S3 Since it is impossible to account for all confounders within the prediction models, we recommend basing the selection mainly on population (healthy, diseases, athletes), age and sex Some of the available prediction models are activity-specific. However, continuous HR measures during free-living conditions typically include a variety of activities. We recommend models that are specific to walking and/or running since this has been shown to yield the highest values of HR_max_ for the general population	Report prediction equation and justification

NB: in longitudinal study designs lasting several years, HR_max_ should be updated regularly at pre-determined time points

HR, heart rate; HR_max_, maximal heart rate

**Table 6:** Proposed protocol for the assessment of nocturnal and/or resting HR

Methodological domains	Methodological variables	Protocol considerations	Reporting considerations
Assessment of nocturnal HR	Preparation	Participants should be informed about the measurement procedures Avoid assessment of nocturnal HR during periods of psychological and/or physical overload (i.e. acute intense exercise) and acute sickness (e.g. flu) Avoid meals and other substances (e.g. caffeine) for 3 h prior to reported bedtime If medically possible, avoid transient medications (e.g. NSAID’s, stimulants, antihistamines, antibiotics, cold medication) for at least 24 h before testing or longer if necessary owing to the half-life of the drug. Medication that is prescribed chronically should be continued as usual During the period of nocturnal HR recording, diaries on physical activity (including exercise) should be kept	Pre-test standardisation should be reported Report physical activity throughout the days of nocturnal HR assessment
	Data collection	Assure that the wearable has been worn during bedtime. Use questionnaires and sleep diaries to control for bedtime If bedtime was not reported, visual inspection of the data is required to verify the device was indeed worn during bedtime (see Data analysis below for details) Wearables do provide information on sleep onset and quality. However, data quality and validity are often not scientifically tested. We suggest not to use those wearable-derived data but rather refer to the two approaches above Select the highest possible sampling rate Since accelerometer-derived data showed that 4 days are sufficient to estimate the physical activity levels of an entire week [11], data should be collected for a minimum of 4 and optimally 7 days	Report the used device and sampling rate (including the firmware) Report whether bedtime was documented and/or controlled Report possible concerns related to sleep quality and well-being
	Data analysis	Use 2-h rolling averages for the duration of reported bedtime. Select the lowest value as nocturnal HR In case bedtime was not reported but the device was worn, 2-h rolling averages should be calculated on the basis of the visually confirmed bedtime NB: some devices may adjust the sampling rate based on the activity performed (e.g. providing data every 5 s up to several minutes). It is advised to choose a device that uses a constant high sampling rate. However, in any case, information on the sampling rate has to be obtained prior to selecting a device. Data points that are within the reported sampling rate range (according to the manufacturer guidelines) should be imputed by using a last value carried forward method on the asis of the highest sampling rate. Missing data (i.e. defined as deviations from a reported sampling rate range) should be excluded from the analysis	Report software/application version for data processing (including download and analysis) Report mean nocturnal HR
Assessment of resting HR	Test preparation	Assessment of resting HR is only recommended in cases where nocturnal HR data are not available (i.e. the device was not worn during bedtime) Participants should be informed about the testing procedures and preparations. Sufficient sleep (i.e. 7–9 h) should be assured prior to the scheduled testing Habitual food and caffeine intake is of no concern Participants using regular medication that affects cardiovascular function (e.g. beta blockers) should be asked to continue intake as usual Avoid transient medications (e.g. NSAID’s, stimulants, antihistamines, antibiotics, cold medication) for at least 24 h before testing or longer if necessary owing to the half-life of the drug Participants should refrain from intense physical activity 48 h prior to the maximal test Participants should avoid any type of activity immediately prior to the testing, including commuting to the lab	Report pre-test standardisation
	Test standardisation	Testing should be performed in standardised ambient conditions (temperature 20–22 °C, humidity < 60%) Ambient light conditions are acceptable; however, we recommend avoiding any sources of unnatural light We recommend the test to be performed in close proximity to awakening. As such, the preferred time of the test should be the early morning Use a 12-lead ECG or chest strap that has been shown to have an excellent agreement with gold standard data to obtain HR. An overview on suitable devices is presented elsewhere [4]	Report ambient conditions, time of day and the device used for HR recording (including the sampling rate and version of the firmware)
	Testing procedure	We recommend resting HR to be assessed in supine position for a duration of 10 min As long as a validated ECG or chest strap is used for the assessment of resting HR, sampling rate is not of concern	Report any deviations from the recommended protocol
	Data analysis	We recommend data be analysed by rolling averages over 15–30 s. The lowest rolling average should be considered as resting HR NB: in cases where data are provided beat-by-beat, conversion to HR data for every second is required	Report software/application version for data processing (including download and analysis) Report resting HR
Alternative resting HR estimation	Data collection	Assure that the wearable has been worn during day time (i.e. from 07:00 am to 10:00 pm) Visual inspection of the data is required to verify the device was indeed worn during daytime (see Data analysis below for details) Select the highest possible sampling rate Since accelerometer-derived data showed that 4 days are sufficient to estimate the physical activity levels of an entire week [11], data should be collected for a minimum of 4 and optimally 7 days	Report the used device and sampling rate (including the firmware) Report factors that may have interfered with an accurate resting HR estimation
	Data analysis	Use a fixed rolling average to find the window for the lowest HR throughout the day. Chose the length of that window on the basis of sampling rate. Since sampling rates tend to vary between few seconds and up to 10 min, the recommended length should be between 5 and 15 min NB: some devices may adjust the sampling rate based on the activity performed (e.g. providing data every 5 s up to several minutes). It is advised to choose a device that uses a constant high sampling rate. However, in any case, information on the sampling rate has to be obtained prior to selecting a device. Data points that are within the reported sampling rate range (according to the manufacturer guidelines) should be imputed by using a last value carried forward method on the basis of the highest sampling rate. Missing data (i.e. defined as deviations from a reported sampling rate range) should be excluded from the analysis	Report software/application version for data processing (including download and analysis) Report mean resting HR

NB: nocturnal HR estimation is recommended as long as HR was recorded throughout the reported bedtime. If that is not the case, resting HR should be assessed. If both nocturnal and resting HR are not available, we propose an alternative procedure as outlined in Table 7. For intra- and inter-individual comparisons, the same approach needs to be applied (i.e. nocturnal or resting HR should be used). In longitudinal study designs, nocturnal and resting HR should be updated regularly at pre-determined time points

HR, heart rate

Irrespective of whether a prospective data collection is planned, or existing data are to be analysed retrospectively, we recommend that HR_max_ is assessed through a standardised protocol (Table 5). However, in case the direct assessment of HR_max_ is not possible, e.g. owing to medical reasons, HR_max_ may be predicted on the basis of existing models. Since studies that evaluated the validity of HR_max_ equations revealed that commonly used universal age-based prediction equations, such as Fox [35] or Tanaka et al. [112], tend to over- or underestimate the HR_max_ when applied to specific populations, it is recommended to choose an equation or model that best represents the target population. An overview on available formulas is presented in online supplementary data Tables S2a–c.

As outlined above, using fixed anchors such as the % of HRmax may lead to inconsistencies in deriving distinct HR zones. Therefore, we suggest utilizing the HRR for quantification of individual physical activity. Consequently, it is necessary to assess nocturnal or resting HR. The aim is to define the lowest physiological HR for a given individual. Thus, we recommend assessing nocturnal HR through continuous HR recordings. Only in cases where overnight data are not available, e.g. owing to discomfort that is often experienced by individuals wearing wearable technology while sleeping, should resting HR be assessed. For recommendations on how to assess nocturnal/resting HR, please refer to online supplementary data, Table 7.

**Table 7:** Proposed protocol for determination of heart rate-based individual activity profiles. The selection of an appropriate method should be based on the specific purpose

Methodological domains	Protocol considerations	Reporting considerations
Determining individual activity profiles	Since studies on accelerometer-derived data showed that 4 days are sufficient to estimate the physical activity levels of an entire week [11], we recommend to include the mean of at least 4 full days in order to determine activity profiles for a given week (i.e. when comparing activity levels in longitudinal study designs). A valid day should include a minimum of 10 h [11] of valid data during waking hours or 16 h [9] over the entire 24-h-cycle We recommend to determine individual activity profiles, using HRR. HRR should be calculated as follows: HRR = HR_max_ − nocturnal HR In case nocturnal HR is not available, the assessed resting HR should be used for calculation of HRR. However, for intra- and inter-individual comparisons, the same procedure is required If the purpose is to assess adherence to common physical activity guidelines or an intensity classification based on traditional approaches is desired, HR zones as provided by the American College of Sports Medicine [17] may be determined. For details see Table 1 and Sect. 6 If the purpose is to assess inter- or intraindividual changes in activity profiles, we recommend displaying individual activity profiles by the time spent in 10% clusters of the HRR (see Sect. 6), expressed as a percentage (i.e. from 100%). Calculate the activity score based on the mean of HRR counts per 10% cluster (see Sect. 6 for details) NB: Some devices may adjust the sampling rate on the basis of the activity performed (e.g. providing data every 5 s up to several minutes). It is advised to choose a device that uses a constant high sampling rate. However, in any case, information on the sampling rate has to be obtained prior to selecting a device. Data points that are within the reported sampling rate range (according to the manufacturer guidelines) should be imputed by using a last value carried forward method on the basis of the highest sampling rate. Missing data (i.e. defined as deviations from a reported sampling rate range) should be excluded from the analysis Furthermore, often night-time is used to charge the batteries of wearables but this period may include critical data (e.g. for the assessment of nocturnal HR). Depending on the aim of the study, participants should be instructed on preferred time slots for battery charging to not miss important data	Report software/application version for data processing (including download and analysis) Report actual time of data analysed (mean number of valid days) If HR-zones are calculated, report time spent in each HR-zone (and indicate the intensity cut-offs used) or in each 10% cluster If desired, additionally report the activity score as outlined in the main manuscript (see Sect. 6)

HR, heart rate

The selection of the method applied to quantify physical activity by HRR should be aligned with the individual research question. Adhering to classical definitions as e.g. provided by the ACSM may allow assessment of adherence to common physical activity guidelines and may also facilitate cautious comparisons to studies that have used accelerometery. However, the physiological inconsistencies underlying these thresholds need to be acknowledged. Considering the limitations that has been brought forward concerning intensity zones based on fixed absolute or relative anchors (see special considerations for determining HR-zones in the section “HR-Zone Determination”), we do feel it is not always desirable to actually report time in specific zones that are linked to metabolic factors but rather provide an individual profile/distribution for each participant. We recommend this to be obtained from HRR, and it can be reported as time spent in arbitrary 10% clusters. Detailed recommendations for the calculation of activity profiles are presented in Table 6. Alternatively, the continuous HR data can be clustered according to the standard intensity zones, for example, as defined by the ACSM: very light, light, moderate, vigorous and near to maximal/maximal exercise intensities (Table 1).

## HR-Metric Calculation and Reporting

**Fig. 2:**
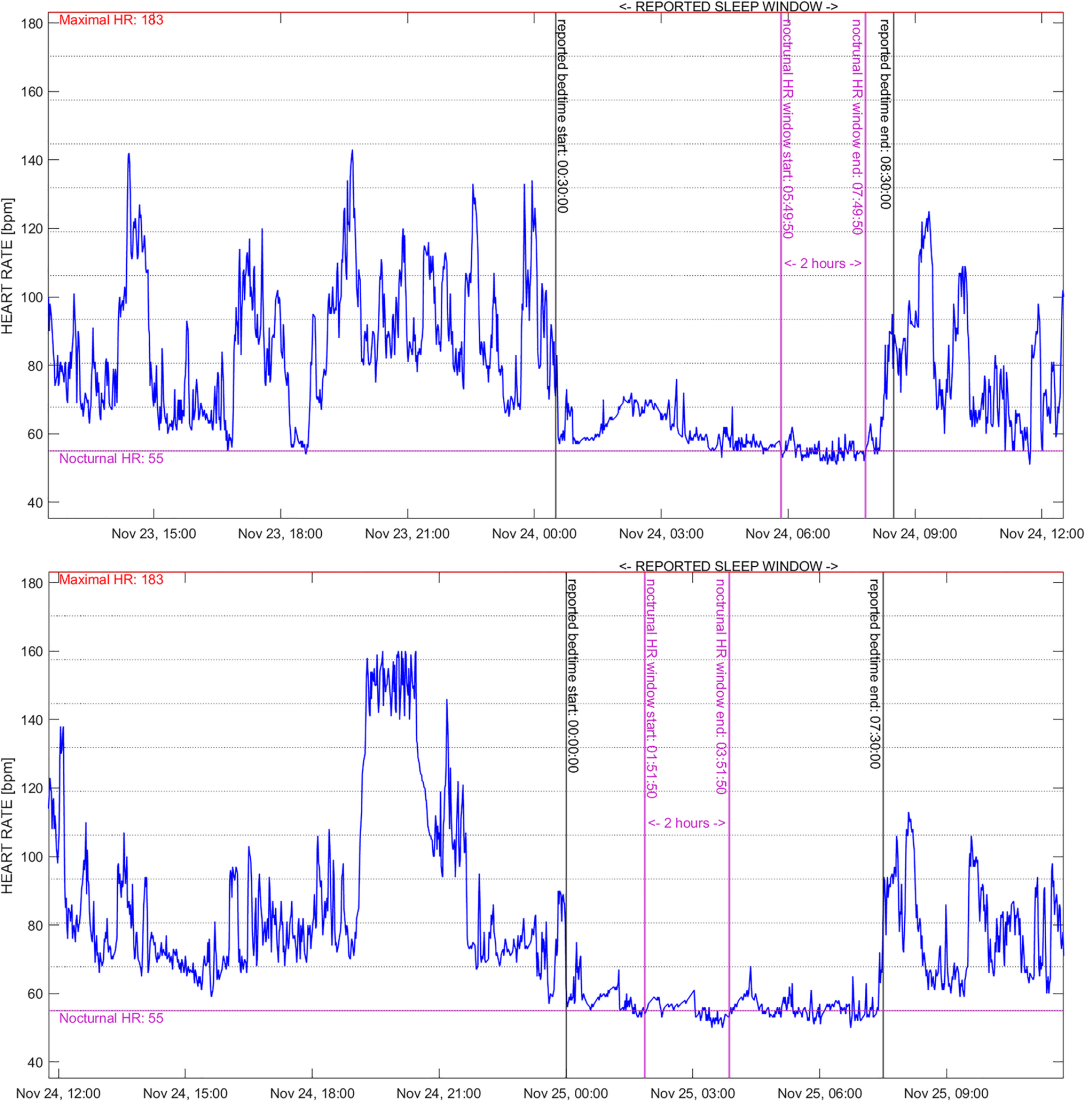
Continuous free-living heart rate (HR) over 24 h illustrating a less (upper plot) and a more (lower plot) active day

On the basis of the considerations discussed above, this section provides a simplified best-practice example of HR-metric calculation for free-living physical activity profiles. The data represent 24-h of an active and inactive day of the same individual (Fig. 2). Data were collected using a Garmin Vivoactive^®^ 4 smartwatch. The sampling interval was 1 min throughout each 24-h data collection. For nocturnal HR assessment, bedtime and time of awakening were reported by the participant in a sleep diary. HR_max_ was assessed during a graded exercise test to exhaustion on a treadmill using the Firstbeat Bodyguard^®^ 2 2-lead ECG. HR_max_, nocturnal HR and activity profiles were calculated using MATLAB (R2023a, Mathworks, Inc., USA). Importantly, with this example we are not aiming to provide guidance on data handling (i.e. statistical processing, including handling of missing data) but rather illustrate in a simplified manner the necessary steps described in this paper (i.e. HR_max_ assessment, nocturnal HR assessment and HR-Zones determination) to profile physical activity by free-living HR.

HR_max_ assessment:

1. HR_max_ was calculated by converting the beat-by-beat HR data provided by the Firstbeat Bodyguard^®^ 2 to beats per minute via the following formula:
  $$\text{HR }\left[\text{bpm}\right]=60\div \text{RR Interval }[\text{sec}] .$$
2. The highest 30-s rolling average was considered HR_max_. HR_max_ was defined as 183 bpm (Fig. 2).

Nocturnal HR assessment:

1. Nocturnal HR was assessed by using 2-h rolling averages to detect the lowest mean HR during the reported sleeping window (Fig. 2). Nocturnal HR was thus determined as 55 bpm.

**Fig. 3:**
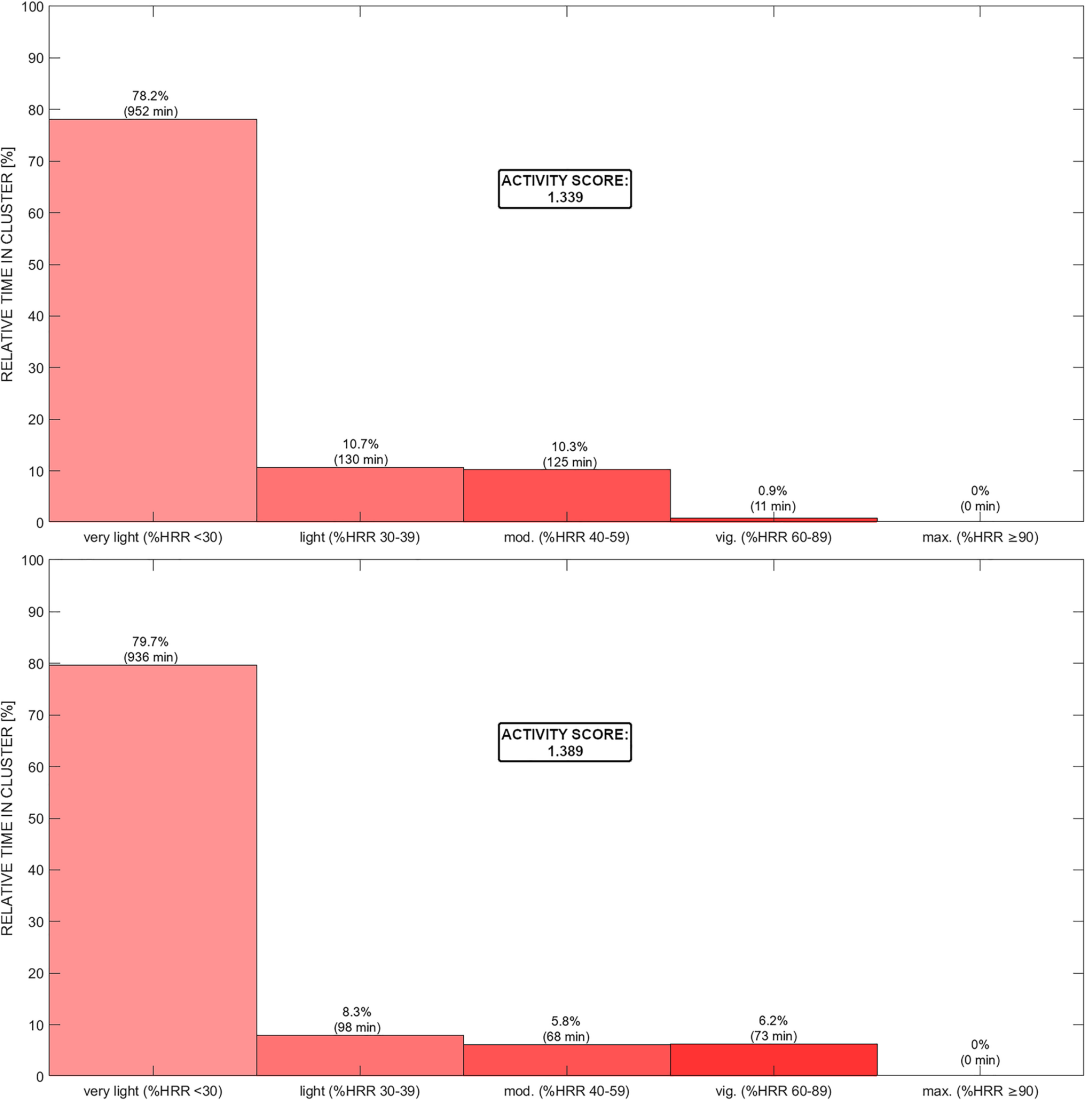
Free-living heart rate (HR) over 24 h illustrating a less (upper plot) and a more (lower plot) active day on the basis of HR reserve (HRR) and clustered according to the common guidelines provided by the American College of Sports Medicine. NB: numbers above the bins indicate the relative and absolute time spent in each zone

**Fig. 4:**
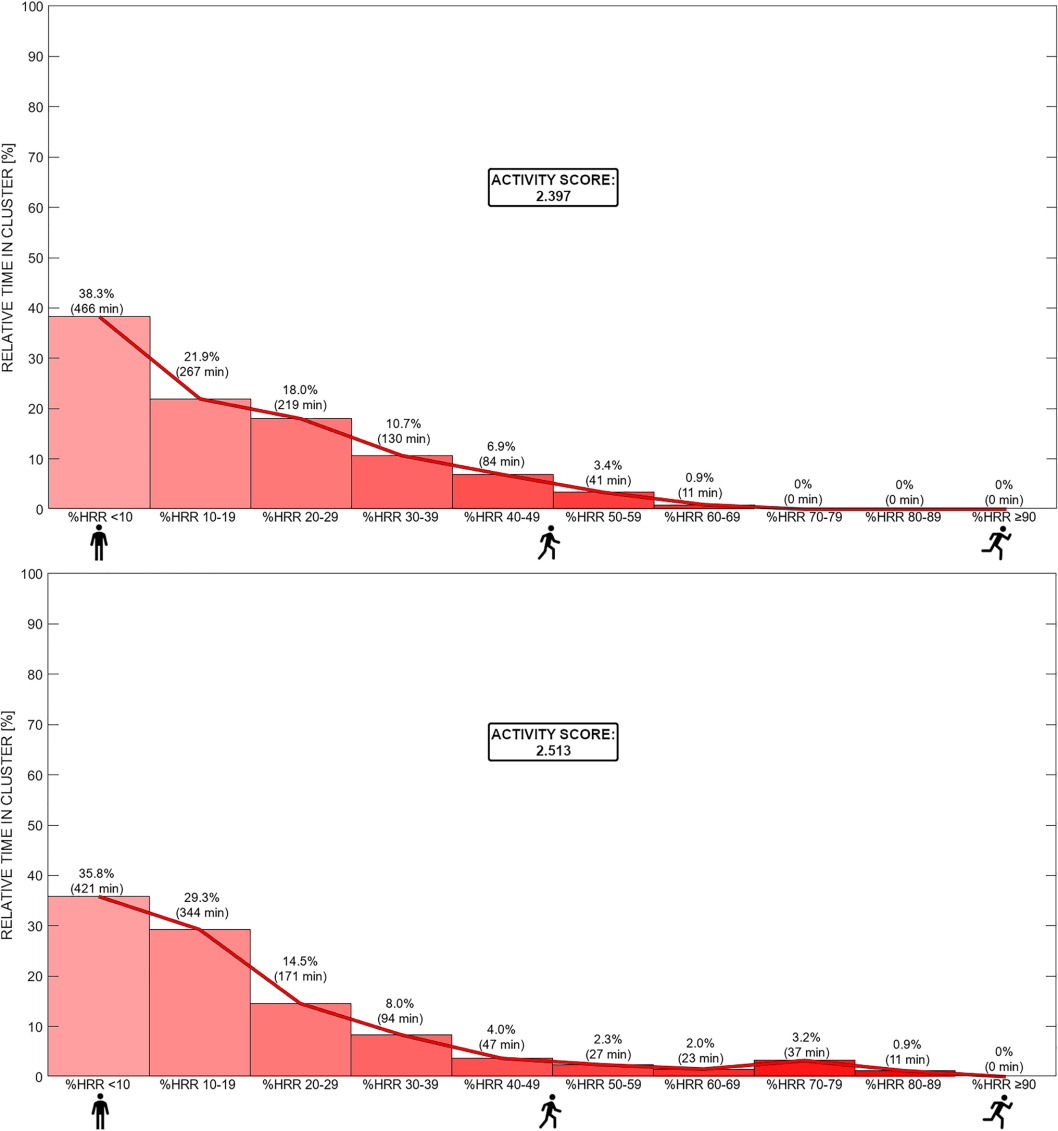
Free-living heart rate (HR) over 24 h illustrating a less (upper plot) and a more (lower plot) active day, displayed in 10% clusters based on HR reserve (HR) (Cluster 1: 0–10% HRR, […], Cluster 10: 91–100% HHR). The activity score was calculated to quantify overall physical activity. NB: numbers above the bins indicate the relative and absolute time spent in each cluster

**Fig. 5:**
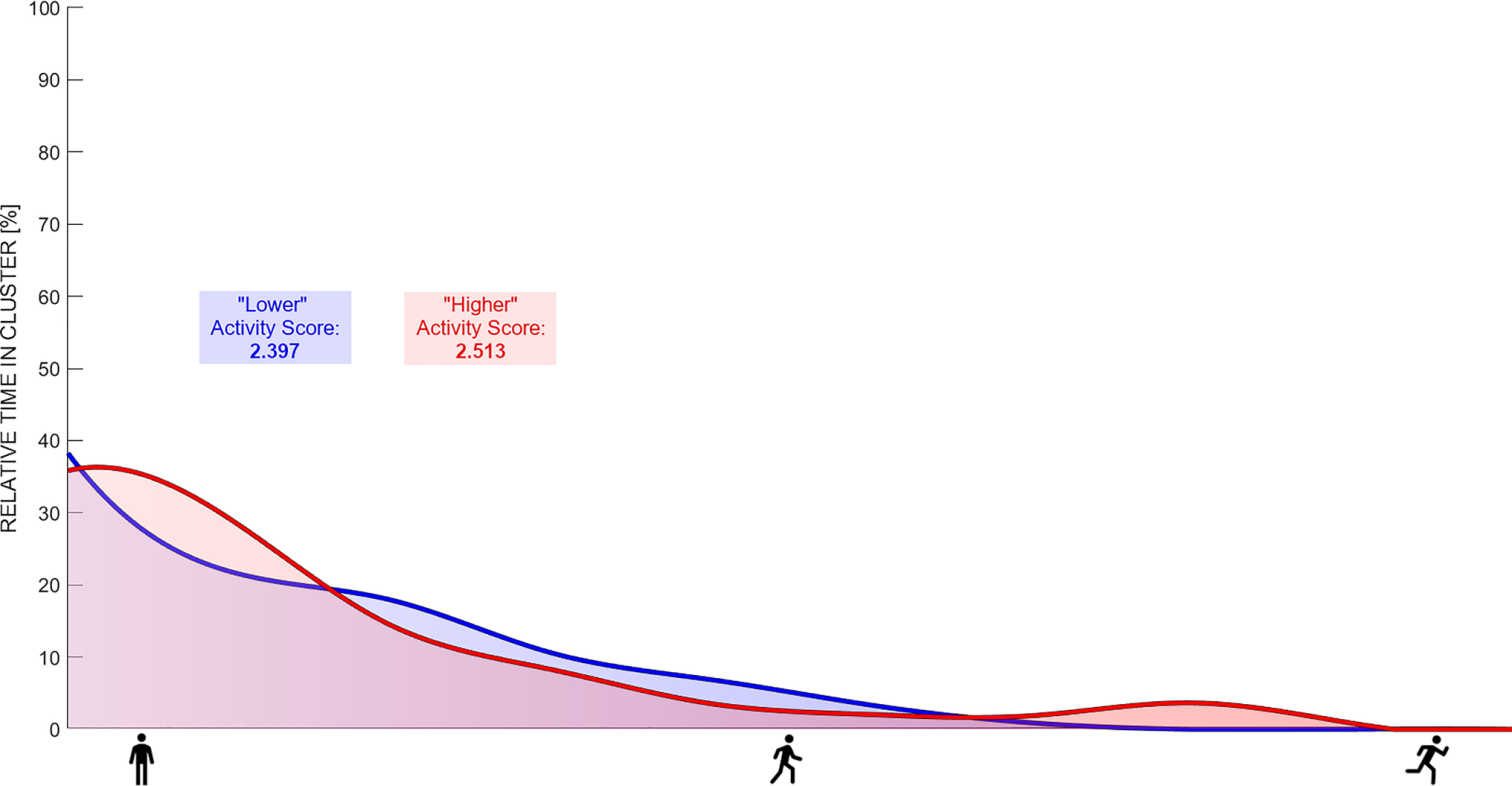
Free-living heart rate (HR) over 24 h illustrating a less (blue) and more (red) active day, displayed as a smoothed curve for a better comparability with the plots shown in Fig. 4. The activity score was calculated to quantify overall physical activity

Activity profiling:

1. Missing data were determined by calculating the difference between the expected number of data points for a complete 24-h measurement on the basis of the sampling interval of 1 min compared with the actual number of data points available.
2. The measurement was considered a valid day because more than 16 h of data were available (Fig. 2).
3. HRR was defined as 128 bpm. The physical activity distribution was displayed on the basis of the recommendations provided by ACSM (Fig. 3) as well as in 10% clusters (Fig. 4). The time spent in each zone or cluster (expressed in minutes or % of the total time) should be reported.
4. If desired for easier comparison or quantification of left–right shifts in the activity profile (based on the research question), an activity score may be calculated as follows:
  $$\text{Activity Score}=\frac{\sum_{i=1}^{n}(\text{time in cluste}{\text{r}}_{\text{i}}\times \text{i}) }{\text{total time}}$$
  *n* = total number of clusters; *i* = number of the specific cluster (e.g. 1–10)
5. For a user-friendly illustration, the activity profile may be smoothed and presented as the physical activity profile (line), and different days could be presented with different colours for instance (Fig. 5).

## Discussion and Future Perspectives

Traditionally, free-living physical activity is assessed by accelerometer data, for which an abundancy of methodological papers is available [9–12, 113, 114]. However, while accelerometer data reflect an external load, HR is considered an internal (often referred to as a relative) measure of intensity. Therefore, HR data have typically been used to evaluate the intensity distribution of single exercise sessions in an attempt to optimize exercise prescription in recreational and elite sport settings. However, since the purpose for tracking individual training sessions differs from that of assessing free-living physical activity, existing theories and recommendations on the use of HR for exercise prescription may also not be appropriate for the quantification of free-living activity. Therefore, we feel the guidelines provided within this paper are timely and primarily aimed at facilitating the comparison of free-living activity data derived from continuous HR measures.

Nevertheless, these guidelines may also be utilized by manufacturers. In fact, our grey-literature search on user manuals and promotional materials of leading companies clearly revealed that the utilization of free-living data for the profiling of physical activity is not yet common and uniform (Table 3). While it is likely that such data may be incorporated into algorithms that provide other measures, such as energy expenditure, only few manufacturers actually visualize activity patterns on the basis of HR. Therefore, we also aim to encourage the use of continuous HR measures as an alternative to sole accelerometer-based data.

There are a few limitations that need to be addressed. First, any of the suggested approaches require access to continuous HR data (as opposed to summary data), which is currently granted only by a minority of manufacturers. Furthermore, the sampling rate may vary considerably between manufacturers and wearable models. In an attempt to save battery life, typically the sampling rate is reduced or automatically adjusted on the basis of the type of activity, affecting data quality. In this context, it is important to bear in mind that HR kinetics typically react slower to activity changes [115, 116] compared with the immediate response of acceleration measures and, therefore, naturally lower sampling rates may be sufficient for HR data. However, an appropriate resolution is needed to minimize the shift to lower HR. Furthermore, for a statistically sound analysis an individual estimation of the number of bins would need to be carried out. However, this would again lead to heterogeneous reporting and, therefore, hinder the comparisons between HR data derived from wearables with different sampling rates and would not help to overcome differences in sampling frequency. In line with this, data processing (including the treatment of missing data) requires special attention but was beyond the scope of this paper.

Collectively, manufacturers are encouraged to further improve free-living HR data quality. This also includes transparent reporting on actual sampling rates. Interestingly, attempts are in place with third-party solutions that allow the set up of customized sampling rates (e.g. the fitrockr software that collaborates with Garmin [117]), allowing access to the continuous HR data as opposed to summary data typically displayed on the user surface of the devices. However, while it is expected that this will provide new horizons for the consistent use of free-living HR data to quantify individual activity patterns, this will also require further advances in analytical approaches. Examples for assessing the accurate distribution of data collected with very high sampling rates are provided by multivariate/functional data analysis for accelerometer data [12] but the application to HR data requires further studies.

## Conclusion and Practical Applications

Combining the information retrieved through a scoping review, grey literature search and the a priori knowledge of the INTERLIVE^®^-network, we provided detailed recommendations for the analysis of free-living HR data to derive individual physical activity profiles obtained by wearables. In addition, this article provides recommendations on how to best measure or predict maximal HR and basal/nocturnal/resting HR.

Since it is well-known that higher levels of physical activity at any intensity and reduced sedentary time are associated with substantial reductions in health risks and premature mortality [108, 118], it is of utmost importance to advance on feasible methods to quantify physical activity and reinforce adherence to physical activity and clinical guidelines. In this context, objective data provided by wearable technology open up new opportunities. Especially HR as an internal measure of activity intensity may overcome some of the limitations related to acceleration-derived physical activity, and may therefore be used as an alternative or complementary method. In this article, we provide a harmonized analytical approach for evaluating physical activity patterns in clinical practice as well as fitness and health settings by free-living HR recordings. Specifically, these recommendations may be applied to various research questions, including randomized controlled trials assessing changes in physical activity patterns, cross-sectional analysis comparing physical activity of distinct populations as well as longitudinal observational studies. While these guidelines are directly useful for researchers and manufacturers, end-users may also benefit, being better informed and empowered to understand and use the HR-information that can be derived from their wearables.

## Supplementary Information

Below is the link to the electronic supplementary material.

Supplementary file1 (DOCX 228 KB)

## Declarations

### Data availability

All underlying data can be found in the online supplementary data.
