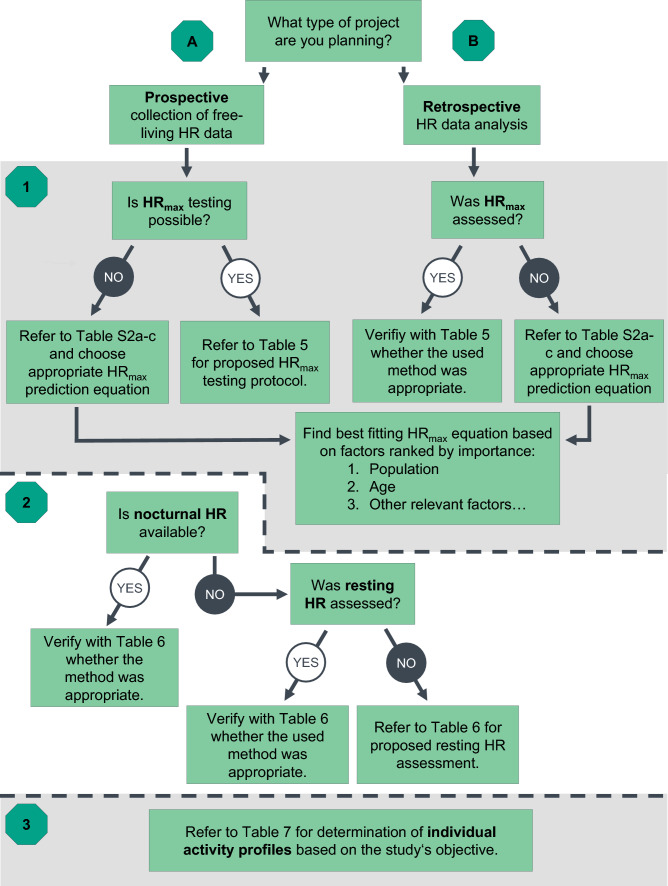# Correction to: Using Free-Living Heart Rate Data as an Objective Method to Assess Physical Activity: A Scoping Review and Recommendations by the INTERLIVE-Network Targeting Consumer Wearables

**DOI:** 10.1007/s40279-025-02329-9

**Published:** 2025-10-23

**Authors:** Moritz Schumann, Joshua F. Feuerbacher, Lars Heinrich, Marcos Olvera-Rojas, Alessandro Sclafani, Jan Christian Brønd, Anders Grøntved, Brian Caulfield, Ulf Ekelund, Wilhelm Bloch, Sulin Cheng, Luis B. Sardinha, Francisco B. Ortega

**Affiliations:** 1 Department of Molecular and Cellular Sports Medicine, German Sport University, Cologne, Germany; 2 Department of Sports Medicine and Exercise Therapy, Chemnitz University of Technology, Chemnitz, Germany; 3 Department of Physical Education and Sports, Faculty of Sport Sciences, Sport and Health University Research Institute (iMUDS), University of Granada, Granada, Spain; 4 Department of Sports Science and Clinical Biomechanics, University of Southern Denmark, Odense C, Denmark; 5 Insight Centre for Data Analytics, University College Dublin, Dublin, Ireland; 6 Department of Sport Medicine, Norwegian School of Sport Sciences, Oslo, Norway; 7 Department of Chronic Diseases, Norwegian Institute of Public Health, Oslo, Norway; 8 Faculty of Sport and Health Sciences, University of Jyväskylä, Jyväskylä, Finland; 9 Exercise, Health and Technology Centre, Department of Physical Education, Shanghai, Jiao Tong University, Shanghai, China; 10 Exercise and Health Laboratory, CIPER, Faculdade de Motricidade Humana, Universidade de Lisboa, Lisbon, Portugal; 11 CIBER de Fisiopatología de La Obesidad y Nutrición (CIBEROBN), Instituto de Salud Carlos III, Granada, Spain

**Correction to: Sports Medicine (2025) 55:275–300** 10.1007/s40279-024-02159-1

In the original article, Fig. 1 appeared incorrectly by referring to Tables in the supplementary material (Tables S5a–c). However, during proofing these Tables were moved to the main manuscript (Tables 5–7). As a result, Fig. 1 has now been corrected in the original publication. For completeness and transparency, both the incorrect and correct versions are displayed below.

Incorrect Fig. 1
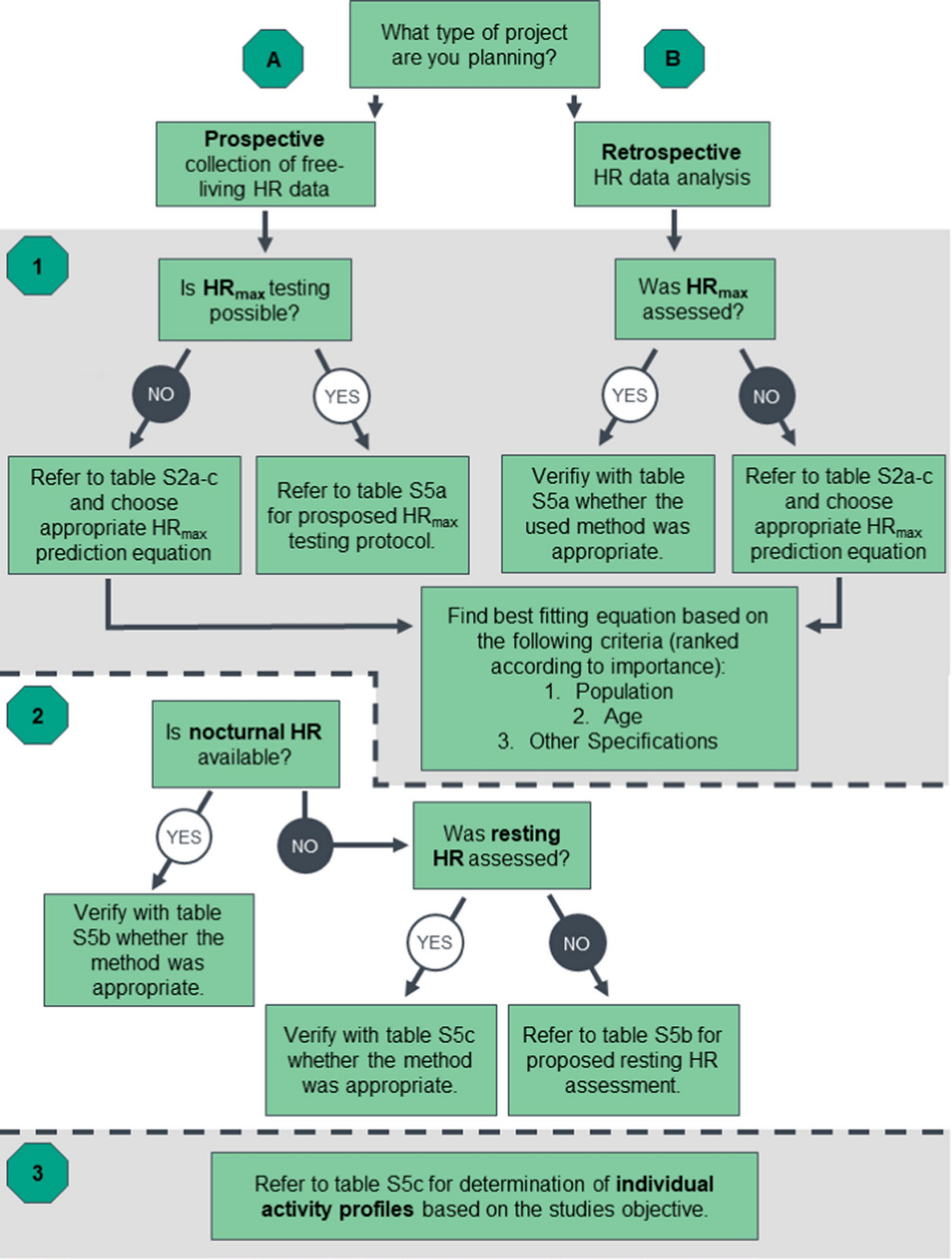


Correct Fig. 1