# Supplementary material for: Using Free-Living Heart Rate Data as an Objective Method to Assess Physical Activity: A Scoping Review and Recommendations by the INTERLIVE-Network Targeting Consumer Wearables
Source: Sports Med. 2025 Feb 2;55(2):275–300. doi: 10.1007/s40279-024-02159-1 (PMC11946962; doi:10.1007/s40279-024-02159-1)
Supplement: Supplementary file 1 — Supplementary file1 (DOCX 228 KB) [file 40279_2024_2159_MOESM1_ESM.docx]

**Online supplementary data**

**Table S1** Search Strings used for the individual data bases.

1. **“HR_max_”**

*PubMed/MEDLINE:*

(heart rate[MeSH Terms]) AND ((“maximal heart rate”[All Fields ]) OR (“maximum heart rate”[All Fields ])) AND ((prediction [All Fields]) OR (estimation[All Fields]) OR (equation[All Fields]))

*ISI Web of Knwoledge:*

ALL= ((“heart rate”)) AND ((“maximal heart rate”) OR (“maximum heart rate”)) AND ((“prediction”) OR (“estimation”) OR (“equation”))

*SPORTDiscus:*

ALL= ((“heart rate”)) AND ((“maximal heart rate”) OR (“maximum heart rate”)) AND ((“prediction”) OR (“estimation”) OR (“equation”))

1. **“Resting HR”**

*PubMed/MEDLINE:*

(heart rate[MeSH Terms]) AND ((“resting heart rate”[All Fields]) OR (“basal heart rate”[All Fields]))

*ISI Web of Knwoledge:*

ALL= (“heart rate”) AND ((“resting heart rate”) OR (“basal heart rate”))

*SPORTDiscus:*

ALL= (“heart rate”) AND ((“resting heart rate”) OR (“basal heart rate”))

1. **“HR-based intensity zones”**

*PubMed/MEDLINE:*

(heart rate[MeSH Terms]) AND ((“intensity zones”[All Fields]) OR (“intensity distribution”[All Fields]) OR (“training intensity”[All Fields]))

*ISI Web of Knwoledge:*

ALL= (“heart rate”) AND ((“intensity zones”) OR (“intensity distribution”) OR (“training intensity”))

*SPORTDiscus:*

ALL= (“heart rate”) AND ((“intensity zones”) OR (“intensity distribution”) OR (“training intensity”))


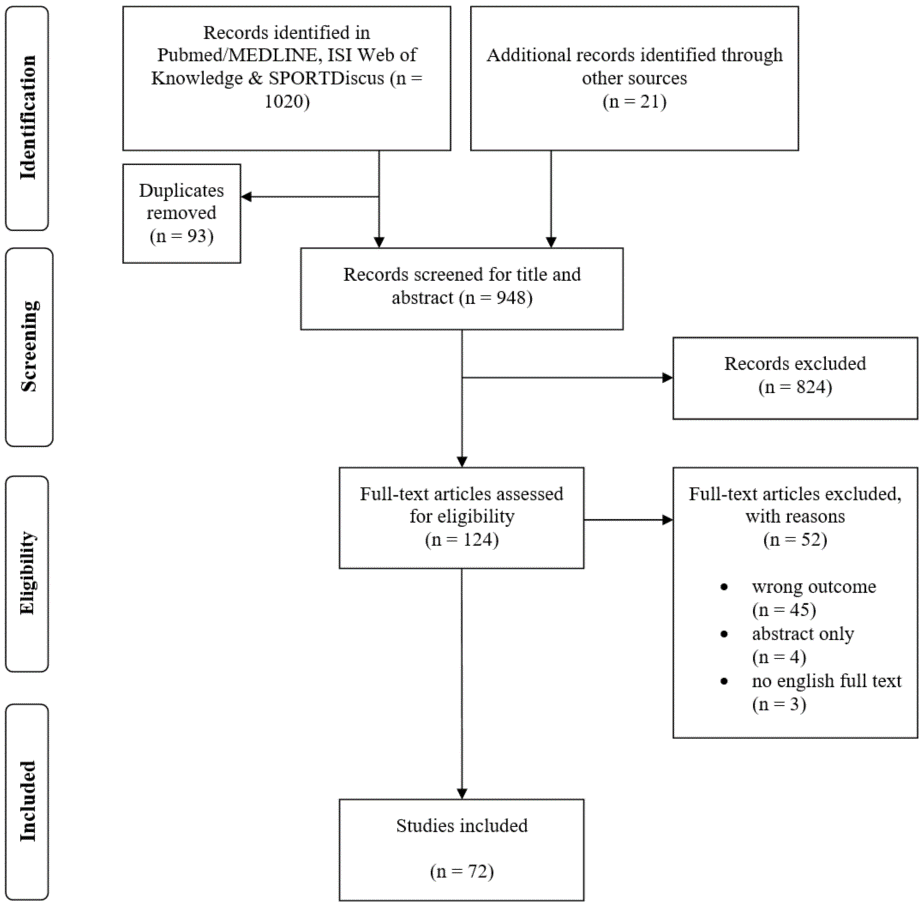


**Figure S1** Flow chart of the search process and the study selection for studies that discussed and/or aimed at assessing models that predict or estimate maximal heart rate (HR_max_).


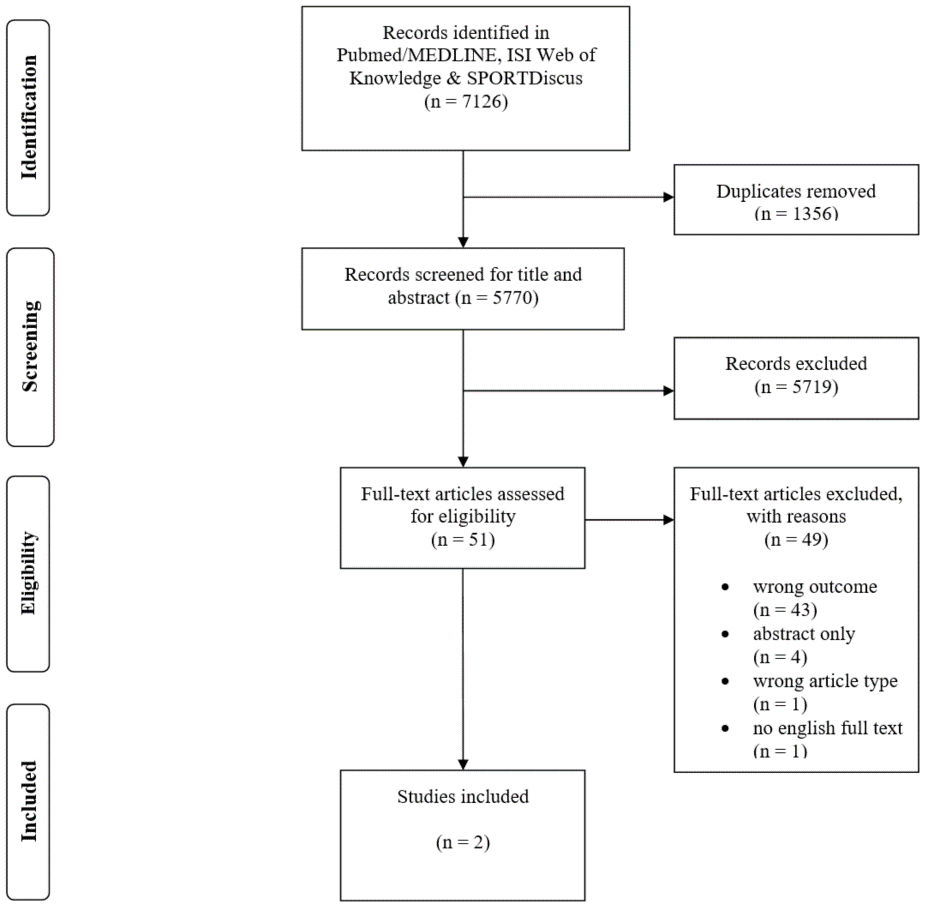


**Figure S2** Flow chart of the search process and the study selection for studies that discussed and/or aimed at estimating basal/resting heart rate.


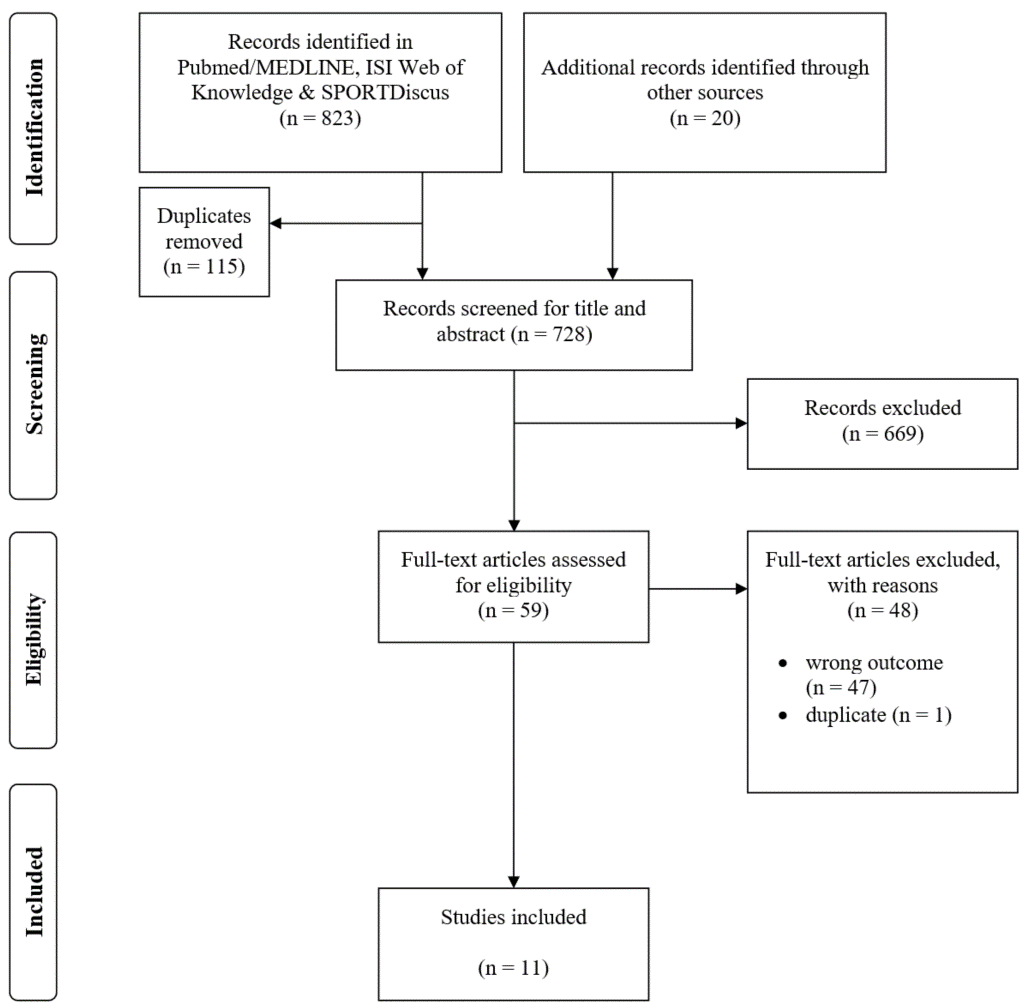


**Figure S3** Flow chart of the search process and the study selection for studies that discussed and/or aimed at assessing heart rate-zones.

**Table S2a** Maximal heart rate (HR_max)_ prediction equations for healthy populations.

| **Author** | **Year** | **Population** | **N** | $\bar{\text{Age}}$ **± SD (range)** | **Equation** |
| --- | --- | --- | --- | --- | --- |
| Arena et al. | 2016 | Healthy (male & female adults) | 4796 | 43 ± 12 | 209.3 - 0.72 × age |
| Astrand et al. | 1952 | Healthy (male & female children & adults) | 225 | 4 - 33 | 216 - 0.84 × age |
| Engels et al. | 1998 | Healthy (male & female adults) | 205 | m: 38.04 ± 12.80 f: 35.14 ± 10.20 | 213.6 - 0.65 × age |
| Fairbarn et al. | 1994 | Healthy (female adults) | 120 | 20 - 80 | 207 - 0.78 × age |
| Fairbarn et al. | 1994 | Healthy (male adults) | 111 | 20 - 80 | 209 - 0.86 × age |
| Fernhall et al. | 2001 | Healthy (male & female, children & adults) | 296 | 31.1 ± 9.3 (9 - 46) | 205 - 0.64 × age |
| Fox et al. | 1972 | Healthy | N/A | N/A | 220 - age |
| Gellish et al. | 2007 | Healthy (male & female adults) | 132 | 44 ± 9.6 | 207 - 0.7 × age |
| Gellish et al. | 2007 | Healthy (male & female adults) | 132 | 44 ± 9.6 | 192 - 0.007 × age^2^ |
| Gellish et al. | 2007 | Healthy (male & female adults) | 132 | 44 ± 9.6 | 163 + 1.16 × age - 0.018 × age^2^ |
| Graettinger et al. | 1995 | Healthy (male adults) | 114 | 43 ± 13 (10 - 73) | 199 - 0.63 × age |
| Gulati et al. | 2010 | Healthy (female adults) | 5437 | 52 ± 11 (>35) | 206 - 0.88 × age |
| Hill et al. | 2016 | Healthy (elderly adults, upper body exercise) | 20 | 66 ± 6 | (208 - 0.7 × age) - 20 |
| Hill et al. | 2016 | Healthy (young adults, upper body exercise) | 30 | 20 ± 2 | (208 - 0.7 × age) - 15 OR (220 - age) - 20 |
| Hossack et al. | 1982 | Healthy (female adults) | 104 | 50 ± 11.1 (20 - 75) | 206 - 0.597 × age |
| Hossack et al. | 1982 | Healthy (male adults) | 98 | 41.7 ± 9..9 (20 - 75) | 227 - 1.067 × age |
| Inbar et al. | 1994 | Healthy (male adults) | 1424 | 46.7 ± 6.4 (20 - 70) | 205.8 - 0.685 × age |
| Johnson et al. | 1991 | Healthy (submaximal test data) | 100 | N/A | 108.461 + 0.5108 × (HR@ rate of perceived exertion=15) - 0.6570 × (HR @ 8min) + 0.6075 × (HR @10min) - 0.2641 × age |
| Jones et al. | 1985 | Healthy (male & female adults, cycle ergometer) | 100 | 15 - 71 | 202 - 0.72 × age |
| Karavirta et al. | 2008 | Healthy (male adults, submaximal HR data) | 74 | 55.6 ± 7.4 (40 - 67) | 160.633 – 0.603 × age + 0.441 × (HR_HF50%_) [= heart rate variability variable: HR at which the high frequency power declined to 50% of the first stage level] |
| Lester et al. | 1968 | Healthy (male & female adults) | 148 | 42.73 | 205.02 - 0.411 × age |
| Londeree & Moeschberger | 1982 | N/A (male & female) | 18155 | 5 - 81 | ^a^ 206.3 - 0.711 × age |
| Londeree & Moeschberger | 1982 | N/A (male & female) | 18155 | 5 - 81 | ^a^ 202.8 - 0.533 × age - 0.0006 × (age^4^/1000) |
| Londeree & Moeschberger | 1982 | N/A (male & female) | 18155 | 5 - 81 | ^a^ 205.0 - 3.574 × T1 + 8.316 × E - 7.624 × F5 - 0.0004 × age^4^ - 0.624 × age |
| Londeree & Moeschberger | 1982 | N/A (male & female) | 18155 | 5 - 81 | ^a^ 196.7 + 1.986 × C2 + 5.361 × E + 1.490 × F4 + 3.730 × F3 + 4.036 × F2 - 0.0006 × age^4^ - 0.542 × age^2^ |
| Londeree & Moeschberger | 1982 | N/A (male & female) | 18155 | 5 - 81 | ^a^ 199.1 + 0.119 × AEF4 + 0.112 × AE + 6.280 × EF3 + 2.468 × C2 + 3.485 × F2 - 0.0006 × age^4^ - 0.591 × age |
| Londeree & Moeschberger | 1982 | N/A (male & female) | 18155 | 5 - 81 | ^a^ 205.0 - 0.116 × AEF3 - 0.223 × AF5 + 0.210 × AE + 6.876 × EF3 + 2.091 × C2 - 3.310 × T1 - 0.0005 × age^4^ - 0.654 × age |
| Mahon et al. | 2010 | Healthy (male & female, children & adolescents) | 52 | 7 - 17 | 158.4 + 0.44 × (resting HR) + 0.68 × (age) |
| Mahon et al. | 2010 | Healthy (male & female, children & adolsecents) | 52 | 7 - 17 | 166.7 + 0.46 × (resting HR) + 1.16 × (maturity offset^b^) |
| Mazzoleni et al. | 2018 | Healthy (male, young adult, cyclists, submaximal HR data) | 12 | 21.1 ± 2.5 | dynamical system model including age and HR during submaximal test on cycle ergometer |
| Miller et al. | 1993 | Healthy (male & female adults) | 51 | 45.3 ± 1.8 | 217 - 0.85 × age |
| Nes et al. | 2013 | Healthy (male & female adults) | 3320 | 19 - 89 | 211 - 0.64 × age |
| Park et al. | 2022 | Healthy (female adolscents, asian) | N/A | 15 - 24 | 214 - 1.2 x age |
| Park et al. | 2022 | Healthy (female adults, asian) | N/A | 40 - 55 | 190 - 0.4 x age |
| Park et al. | 2022 | Healthy (female children, asian) | N/A | 7 - 14 | 218 - 0.8 x age |
| Park et al. | 2022 | Healthy (female young adults, asian) | N/A | 25 - 39 | 189 - 0.3 x age |
| Park et al. | 2022 | Healthy (female, asian) | 392 | 7 - 55 | 209 - 0.9 x age |
| Park et al. | 2022 | Healthy (male adolsecents, asian) | N/A | 15 - 24 | 214 - 0.8 x age |
| Park et al. | 2022 | Healthy (male adults, asian) | N/A | 40 - 55 | 210 - 0.7 x age |
| Park et al. | 2022 | Healthy (male children, asian) | N/A | 7 - 14 | 220 - age |
| Park et al. | 2022 | Healthy (male young adults, asian) | N/A | 25 - 39 | 212 - 0.8 x age |
| Park et al. | 2022 | Healthy (male, asian) | 280 | 7 - 55 | 219 - age |
| Ricard et al. | 1990 | Healthy (male & female, bicycle) | 193 | 6 - 50 | 205 - 0.687 × age |
| Ricard et al. | 1990 | Healthy (male & female, treadmill) | 193 | 6 - 50 | 209 - 0.587 × age |
| Rodeheffer et al. | 1984 | Healthy (male adults) | 61 | 25 - 79 | 214 - 1.02 × age |
| Schiller et al. | 2001 | Healthy (female adults, caucasian) | 93 | 42 (20 - 75) | 207 - 0.62 × age |
| Schiller et al. | 2001 | Healthy (female adults, hispanic ) | 53 | 46 (20 - 75) | 213.7 - 0.75 × age |
| Shargal et al. | 2015 | Healthy (female) | 7446 | 10 - 80 | 209.273 - 0.804 × age |
| Shargal et al. | 2015 | Healthy (male & female) | 28137 | 10 - 80 | 208.852 - 0.741 × age |
| Shargal et al. | 2015 | Healthy (male) | 20691 | 10 - 80 | 208.609 - 0.716 × age |
| Sheffield et al. | 1978 | Healthy (female adults) | 95 | 38.9 (19 - 69) | 216 - 0.88 × age |
| Tanaka et al. | 2001 | Healthy (male & female adults, active) | N/A | 18 - 81 | 207 - 0.7 × age |
| Tanaka et al. | 2001 | Healthy (male & female adults, endurance trained) | 229 | 18 - 81 | 206 - 0.7 × age |
| Tanaka et al. | 2001 | Healthy (male & female adults, general) | 514 | 18 - 81 | 208 - 0.7 × age |
| Tanaka et al. | 2001 | Healthy (male & female adults, sedentary) | 285 | 18 - 81 | 211 - 0.8 × age |
| Tao et al. | 2021 | Healthy (adults) | 121 | 57.2 ± 6.4 (41 - 71) | 201.77 - 0.88 × age |
| Tao et al. | 2021 | Healthy (adults, submaximal HR data) | 121 | 57.2 ± 6.4 (41 - 71) | 150.46 + 0.43 × resting HR - 0.96 × age + 0.2 × second order heart rate |
| Whaley et al. | 1992 | Healthy (female) | 754 | 14 - 77 | 208.8 - 0.723 × age |
| Whaley et al. | 1992 | Healthy (female) | 754 | 14 - 77 | 204.8 × 0.718 × age + 0.162 × resting HR - 0.105 × (bodyweight (kg)) - 6.2 × smoking status (0 = non-smoker, 1 = smoker) |
| Whaley et al. | 1992 | Healthy (male) | 1256 | 14 - 77 | 213.6 - 0.789 × age |
| Whaley et al. | 1992 | Healthy (male) | 1256 | 14 - 77 | 203.9 × 0.812 × age + 0.76 × resting HR - 0.084 × (bodyweight (kg)) - 4.5 × smoking status (0 = non-smoker, 1 = smoker) |
| Whyte et al. | 2008 | Healthy (female, sedentary) | 56 | 16 - 33 | 221 - 1.09 × age |
| Whyte et al. | 2008 | Healthy (male, sedentary) | 39 | 17 - 35 | 207 - 0.55 × age |

HR, heart rate.

^a^ C#=continent (If European, then C2=1, otherwise C2=0); E=ergometer (If treadmill, then E=1, bicycle then E=O); f #=fitness level (If sedentary, F2=1, otherwise F2=0; if active, then F3=1, otherwise F3=0, if endurance trained, then F4=1, otherwise F4=0; F5 and F6 represent collegiate and national class endurance athletes, respectively); and Type #=type of exercise protocol (If continuous and incremental,then T1=1, otherwise T1=0). HR, heart rate.

^b^predicts the child’s age in years, relative to the estimated age at which he or she attains peak growth height (Mahon et al. 2010)

**Table S2b** HR_max_ prediction equations for athletic populations.

| **Author** | **Year** | **Population** | **N** | $\bar{\text{Age}}$ **± SD (range)** | **Equation** |
| --- | --- | --- | --- | --- | --- |
| Cruz et al. | 2012 | Athletes (adults, cyclists) | 131 | 46 ± 4.98 (40 - 60) | 246.8 - 1.5592 × age |
| Faff et al. | 2006 | Athletes (male & female adults, all ergometers) | 3010 | 16 - 24 | 208.5 - 0.8 × age |
| Faff et al. | 2006 | Athletes (female adults, all ergometers) | 1244 | 16 - 24 | 208.3 - 0.74 × age |
| Faff et al. | 2006 | Athletes (female adults, cycle ergometer) | 291 | 16 - 24 | 211.6 - 0.88 × age |
| Faff et al. | 2006 | Athletes (female adults, kayak ergometer) | 578 | 16 - 24 | 205.1 - 0.68 × age |
| Faff et al. | 2006 | Athletes (female adults, rowing ergometer) | 74 | 16 - 24 | 206.1 - 0.50 × age |
| Faff et al. | 2006 | Athletes (female adults, ski ergometer) | 13 | 16 - 24 | 194.3 - 0.31× age |
| Faff et al. | 2006 | Athletes (female adults, treadmill) | 283 | 16 - 24 | 213.8 - 0.81 × age |
| Faff et al. | 2006 | Athletes (male adults, all ergometers) | 1766 | 16 - 24 | 207.5 - 0.78 × age |
| Faff et al. | 2006 | Athletes (male adults, cycle ergometer) | 293 | 16 - 24 | 204.4 - 0.72 × age |
| Faff et al. | 2006 | Athletes (male adults, kayak ergometer) | 918 | 16 - 24 | 206.7 - 0.81 × age |
| Faff et al. | 2006 | Athletes (male adults, rowing ergometer) | 138 | 16 - 24 | 206.7 - 0.81 × age |
| Faff et al. | 2006 | Athletes (male adults, ski ergometer) | 10 | 16 - 24 | 197.7 - 0.42 × age |
| Faff et al. | 2006 | Athletes (male adults, treadmill) | 400 | 16 - 24 | 209.9 - 0.73 × age |
| Gelbart et al. | 2017 | Athletes (children & adolescent, male & female) | 433 | 13.7 ± 2.1 | 168 + 0.259 × resting HR - 0.156 × (body mass (kg)) + 0.891 × METs + 0.256 × body fat percentage |
| Ghouli et al. | 2023 | Athelets (children & adolescent, male soccer players) | 801 | 11 - 18 | 225.08 – 1.55 × age |
| Lester et al. | 1968 | Athletes (male & female adults) | 42 | 30.24 | 198.19 - 0.411 × age |
| Matabuena et al. | 2019 | Athletes (male & female, children & adults, submaximal HR data) | 360 | 10 - 46 | Functional Regression Model: s(HR0,6) + s(dHR0,6) + s(HR_max_0,6) + s(age) [= derivative of HR in the first six minutes of the treadmill test] |
| Matabuena et al. | 2019 | Athletes (male & female, children & adults) | 360 | 10 - 46 | 209.92 - 0.77 × age |
| Matabuena et al. | 2019 | Athletes (male & female, children & adults) | 360 | 10 - 46 | 206 + 1.167e-02 × age - 4.435e-02 × age^2^ + 6925e-04 × age^3^ |
| Nikolaidis et al. | 2015 | Athletes (general, soccer players) | 320 | 19.7 ± 5.1 (11.3 - 35.8) | 212.3 - 0.75 × age |
| Nikolaidis et al. | 2015 | Athletes (adults, soccer players) | 162 | 23.4 ± 4.6 (18.0 - 35.8) | 213.2 - 0.78 × age |
| Nikolaidis et al. | 2015 | Athletes (children & adolescents, soccer players) | 158 | 15.8 ± 1.5 (11.3 - 18.0) | 223 - 1.44 × age |
| Nikolaidis et al. | 2018 | Athletes (male & female adults, recreational marathon runners) | 180 | 43.2 ± 8.5 | 204.4 - 0.6058 × age |
| Nikolaidis et al. | 2018 | Athletes (female adults, recreational marathon runners) | 32 | 40.3 ± 8.8 | 199.2 - 0.6033 x age |
| Nikolaidis et al. | 2018 | Athletes (male adults, recreational marathon runners) | 148 | 43.9 ± 8.3 | 208 - 0.6632 x age |
| Whyte et al. | 2008 | Athletes (female, elite endurance and sprint/power trained) | 75 | 15 - 34 | 216 - 1.09 × age |
| Whyte et al. | 2008 | Athletes (male, elite endurance and sprint/power trained) | 85 | 15 - 38 | 202 - 0.55 × age |

HR, heart rate

**Table S2c** HR_max_ prediction equations for diseased populations

| **Author** | **Year** | **Population** | **N** | $\bar{\text{Age}}$ **± (range)** | **Equation** |
| --- | --- | --- | --- | --- | --- |
| Brawner et al. | 2004 | Diseased (male & female adults, patients with coronary heart disease not recieving beta blockers) | 128 | 40 - 80 | 200 - 0.92 × age |
| Brawner et al. | 2004 | Diseased (male & female adults, patients with coronary heart disease recieving beta blockers) | 334 | 40 - 80 | 164 - 0.72 x age |
| Casillas et al. | 2015 | Diseased (male & female adults, patients with coronary heart disease, submaximal HR data) | 148 | 59 ± 9 | 130 - 0.6 × age + 0.3 × HR200mFWT [= HR at the end of the 200 m fast-walk test] |
| Godlasky et al. | 2018 | Diseased (male & female adults, coronary artery disease, not recieving beta blockers) | 110 | 63 ± 10 | 200 - 0.79 × age |
| Godlasky et al. | 2018 | Diseased (male & female adults, coronary artery disease, took beta blockers 12 to 24 hours prior to testing) | 155 | 62 ± 11 | 193 - 0.71 × age |
| Godlasky et al. | 2018 | Diseased (male & female adults, coronary artery disease, took beta blockers today) | 72 | 60 ± 12 | 168 - 0.51 × age |
| Hammond et al. | 1983 | Diseased (male adults, coronary heart disease) | 156 | 53.9 (35 - 65) | 209 - age |
| Keteyian | 2012 | Diseased (male & female adults, heart failure patients, taking beta blocker) | 767 | 57 (49–66) | 119 + 0.5 × resting HR - 0.5 × age − (0, for treadmill OR 5, for stationary bike) |
| Magrì et al. | 2022 | Diseased (male & female adults, heart failure patients with reduced ejection fraction) | 3487 | 59 ± 12 | 109 - (0.5 x age) + (0.5 x resting HR) + (0.2 x LVEF) - (5 if haemoglobin < 11 g/dL)] |
| Fernandes Silva et al. | 2012 | Diseased (male & female adults, ischemic heart failure patients receiving beta blockers) | 75 | 46.6 ± 10.6 | 168 - 0.76 × age |
| Sydó et al. | 2014 | Diseased (female adults, cardiovascular disease excluded) | 6748 | 55 ± 9 (40 - 89) | 210 - 0.79 × age |
| Sydó et al. | 2014 | Diseased (male adults, cardiovascular disease excluded) | 12265 | 54 ± 9 (40 - 89) | 221 - 0.95 × age |
| Miller et al. | 1993 | Diseased (male & female adults, obese) | 86 | 42 ± 1 | 200 - 0.48 × age |
| Verschuren et al. | 2011 | Diseased (male & female, children & adolescents, cerebral palsy) | 362 | 6 - 19 | 194 bpm = HR_max_ [did not vary with age] |
| Fernhall et al. | 2001 | Diseased (children & adults, mental retardation, down syndrome) | 276 | 21.8 ± 8.4 (9 - 46) | 210 - (0.56 × age) - (15.5 × (Down Syndrome = 2, No Down Syndrome = 1)) |

HR, heart rate; LVEF, left ventricular ejection fraction

***Table S3*** *Studies identified by the systematic literature search that solely aimed at evaluating the validity of already existing equations.*

| **Author** | **Year** | **Population for which the equation was evaluated** | **N** | $\bar{\text{Age}}$ **± SD (range)** | **Evaluated HR_max_ predicition equation** | | **Conclusion (briefly summarized)** |
| --- | --- | --- | --- | --- | --- | --- | --- |
| Antonacci et al. | 2007 | Athletes (male, soccer players) | 45 | 19.24 | Fox | 220 - age | Fox overestimates HR_max_ compared to the highest HR during competitive matches and the highest HR during a 1000-meter maximal effort tests |
| Berkelmans et al. | 2018 | Athletes (male, basketball players) | 6 | 21.7 ± 4.9 (17 - 29) | Fox | 220 - age | Authors do not recommend to apply any age predicted models to determine HR_max_ when calculating summated-HR-zone outcomes. However, in some individual cases, age-predicted approaches might be valid. |
|  |  |  |  |  | Hossack | 206 - 0.597 × age |  |
|  |  |  |  |  | Tanaka | 208 - 0.7 × age |  |
|  |  |  |  |  | Nikolaidis | 212.3 − 0.75 × age |  |
|  |  |  |  |  | Nes | 211 - 0.64 × age |  |
|  |  |  |  |  | Faff | 209.9 - 0.73 × age |  |
| Boer et al. | 2017 | Diseased (male & female adults, mental retardation, down syndrome) | 36 | 31.7 ± 6.8 (19 - 46) | Fernhall | 210 - (0.56 × age) - (15.5 × (Down syndrome = 2, No Down syndrome = 1)) | Significant difference between directly measured HR_max_ during a maximal effort treadmill test and predicted HR_max._ Measurement bias (+4.7 bpm) and large limits of agreement (+26.7 bpm and -17.4 bpm). The equation was not accurate in this sample. |
| Boone et al. | 2013 | Healthy (male & femlae children & adolsecents) | 145 | 7 - 17 | Fox | 220 - age | The Fox equation overestimated HR_max_ during a maximal incremental test (+25.53 bpm; SD = 14.67). Sex and training level were not related to the HR_max_, independently of whether HR_max_ was predicted or measured. The equation is not recommended for exercise training in children. |
| Branco et al. | 2020 | Athletes (male adults, brazilian jiu jitsu) | 13 | 29.5 ± 5.9 | Fox | 220 - age | Fox and Tanaka overestimated the HR_max_ compared to maximal effort test on a treadmill and a cooper field test. |
|  |  |  |  |  | Tanaka | 208 - 0.7 × age |  |
| Camarda et al. | 2008 | Healthy (male & female adults) | 2047 | 37.1 ± 11.4 (12 - 69) | Fox | 220 - age | HR_max_ values from both equations were significantly higher than those obtained during a maximal graded exercise test on a treadmill. The measured HR_max_ correlated well with prediction equations. |
|  |  |  |  |  | Tanaka | 208 - 0.7 × age |  |
| Cicone et al. | 2019 | Healthy (male & female, children & adolescents) | 648 | < 18 | Fox | 220 - age | The authors do not recommend the Fox equation, as it overestimated HR_max_ by 12.4 bpm and had limits of agreement of ± 16.2 bpm. Tanaka underestimated HR_max_ by 2.7 bpm and had limits of agreement of ± 5.8 bpm. The Tanaka equation produces less mean bias and accounts for more individual variation in HR_max_. |
|  |  |  |  |  | Tanaka | 208 - 0.7 × age |  |
| Cicone et al. | 2018 | Athletes (adolescent male, soccer players) | 30 | 14.6 ± 0.6 | Fox | 220 - age | There were no significant differences between HR_max_ from a maximal effort treadmill test and predicted HR_max_ from the Fox and Nikolaidis equations. The Tanaka and Shargal equations significantly underestimated HR_max_. All 4 equations produced limits of agreement of ±15.0 bpm. The wide limits of agreement suggest that none of the equations adequately account for individual variability in HR_max_. Practitioners should avoid applying these equations in youth athletes and utilize a lab or field-testing protocol to obtain HR_max_. |
|  |  |  |  |  | Tanaka | 208 - 0.7 × age |  |
|  |  |  |  |  | Shargal | 201.104 - 0.326 × age |  |
|  |  |  |  |  | Nikolaidis | 223 - 1.44 × age |  |
| Cleary et al. | 2011 | Healthy (male & female young adults, physically active) | 96 | 22 ± 2.8 (18 - 33) | Fox | 220 - age | The Gellish (192 - 0.007 × age^2^) and Fairbarn equations were the most accurate of the age-predicted HR_max_ equations in a college-age population compared to a criterion HR_max_ measure (30-second Wingate anaerobic cycling test (WAnT) and 2 200-m sprint trials, with each test separated by at least 3 days). Predicted HR_max_ from Fox, Gellish (207 - 0.7 × age), Gellish (163 + 1.16 × age - 0.018 × age^2^), Tanaka, and Hossack were all significantly different from the criterion HR_max_. The Fox equation resulted in overpredicting HR_max_ in 88.5% of the cases. When the use of a direct HR_max_ test is impractical, the Gellish (192 - 0.007 × age^2^) or the Fairbarn equations should be used. |
|  |  | Healthy (male & female young adults, physically active) | 96 | 22 ± 2.8 (18 - 33) | Tanaka | 208 - 0.7 × age |  |
|  |  | Healthy (male & female young adults, physically active) | 96 | 22 ± 2.8 (18 - 33) | Gellish | 207 - 0.7 × age |  |
|  |  | Healthy (male & female young adults, physically active) | 96 | 22 ± 2.8 (18 - 33) | Gellish | 192 - 0.007 × age^2^ |  |
|  |  | Healthy (male & female young adults, physically active) | 96 | 22 ± 2.8 (18 - 33) | Gellish | 163 + 1.16 × age - 0.018 × age^2^ |  |
|  |  | Healthy (female young adults, physically active) | 44 | 22.4 ± 2.8 (18 - 33) | Fairbarn (f) | 207 - 0.78 × age |  |
|  |  | Healthy (male young adults, physically active) | 52 | 21.8 ± 3.3 (18 - 33) | Fairbarn (m) | 209 - 0.86 × age |  |
|  |  | Healthy (female young adults, physically active) | 44 | 22.4 ± 2.8 (18 - 33) | Hossack (f) | 206 - 0.597 × age |  |
|  |  | Healthy (male young adults, physically active) | 52 | 21.8 ± 3.3 (18 - 33) | Hossack (m) | 227 - 1.067 × age |  |
| Dos Reis et al. | 2023 | Diseased (male & female adults, post stroke patients) | 60 | 54 ± 12 | Fox | 220 - age | Bland-Altman plots showed that equations (1-4) and (6) overestimated the HRmax. Equation (6) presented the lower mean difference. The equations developed for non-disabled individuals (1-4) are not adequate to be used in individuals after a stroke. Equation Brawner showed the best results to be used in individuals after stroke; however, it should be used cautiously. |
|  |  |  |  |  | Gellish | 207 - 0.7 × age |  |
|  |  |  |  |  | Tanaka | 208 - 0.7 × age |  |
|  |  |  |  |  | Astrand | 216 - 0.84 × age |  |
|  |  |  |  |  | Brawner | 200 - 0.92 × age |  |
| Esco et al. | 2015 | Athletes (female college athletes) | 30 | 21.5 ± 1.9 | Fox | 220 - age | Fox, Astrand, and Tanaka significantly overestimated HR_max_ on average. The female-specific HR_max_ equations of Fairbarn and Gulati provided the most accurate mean values. However, all of the equations that were analyzed in the study provided large limits of agreement and showed tendencies to overpredict HR_max_. Because of the wide limits of agreement displayed by each equation, the use of age predicted methods for estimating HR_max_ in collegiate female athletes should be performed only with caution. |
|  |  |  |  |  | Tanaka | 208 - 0.7 × age |  |
|  |  |  |  |  | Fairbarn | 201 - 0.63 × age |  |
|  |  |  |  |  | Astrand | 216 - 0.84 × age |  |
|  |  |  |  |  | Gulati | 206 - 0.88 × age |  |
| Franckowiak et al. | 2011 | Diseased (male & female adults, overweight & obese) | 154 | 20 - 60 | Fox | 220 - age | Prediction equations showed close agreement to actual HR_max_, with Tanaka being the most accurate compared to a graded maximal treadmill test. |
|  |  |  |  |  | Tanaka | 208 - 0.7 × age |  |
|  |  |  |  |  | Miller | 200 - 0.5 × age |  |
| Gallagher et al. | 2015 | Healthy (male & female adults, in hypoxic conditions) | 15 | 22 ± 2 | Fox | 220 - age | The HR_max_ was overestimated by all equations compared to the measured HR_max_ during an incremental cycle ergometer test. Researchers should directly measure HR_peak_ whenever possible if it is to be used to prescribe exercise intensities in normobaric hypoxic conditions. |
|  |  |  |  |  | Tanaka | 208 - 0.7 × age |  |
|  |  |  |  |  | Tanaka | 211 - 0.8 × age |  |
|  |  |  |  |  | Tanaka | 207 - 0.7 × age |  |
|  |  |  |  |  | Jones | 202 - 0.72 × age |  |
|  |  |  |  |  | Jones | 210 - 0.65 × age |  |
|  |  |  |  |  | Ricard | 209 - 0.587 × age |  |
|  |  |  |  |  | Ricard | 200 - 0.687 × age |  |
| Garlipp et al. | 2016 | Athletes (male adult, soccer players) | 90 | 22.2 ± 4.4 (16 - 33) | Fox | 220 - age | None of the three available equations (Fox, Tanaka and Nikolaids) provided accurate values of HR_max_ in a sample of professional soccer players compared to a maximal treadmill test. However, the equation that provided the most accurate measured HR_max_ was the Nikolaids equation. |
|  |  |  |  |  | Tanaka | 208 - 0.7 × age |  |
|  |  |  |  |  | Nikolaidis | 223 − 1.44 × age |  |
| Heinzmann-Filho et al. | 2018 | Diseased (male & female adolescents, obese) | 59 | 16.8 ± 1.2 (15 - 18) | Fox | 220 - age | Fox, Tanaka and Gellish were shown to overestimate the measured HR_max_ results in obese adolescents compared to a maximal treadmill test. Only the Miller equation presented similar results with the measured values. |
|  |  |  |  |  | Tanaka | 208 - 0.7 × age |  |
|  |  |  |  |  | Gellish | 207 - 0.7 × age |  |
|  |  |  |  |  | Miller | 200 - 0.5 × age |  |
| Kasiak et al. | 2023 | Atheletes (male & female adults, runners) | 4043 | 33.58 ± 8.12 | Nes | 211 − 0.64 × age | Predicted HRmax was significantly different from that observed in CPET across most models (11 of 13). Among the selected equations, 66% (five of eight for treadmill and three of five for cycle ergometer) underestimated HRmax and 33% (three of eight for treadmill and two of five for cycle ergometer) overestimated HRmax. The lowest accuracy has been noted for the Machado running formula and cycling equation provided by Fairbarn. Prediction models allow for limited precision of HRmax estimation and present inaccuracies. Underestimation of HRmax occurred more often than overestimation. |
|  |  |  |  |  | Machado | 218 − 0.8 × age |  |
|  |  |  |  |  | Tanaka | 208 − 0.7 × age |  |
|  |  |  |  |  | Fox | 220 − age |  |
|  |  |  |  |  | Londeree | 206.3 − 0.711 × age |  |
|  |  |  |  |  | Inbar | 205.8 − 0.685 × age |  |
|  |  |  |  |  | Gellish | 207 − 0.7 × age |  |
|  |  |  |  |  | Arena | 209.3 − 0.72 × age |  |
|  |  | Atheletes (male & female adults, cyclists) | 1268 | 36.88 ± 9.03 | Tanaka | 208 − 0.7 × age |  |
|  |  |  |  |  | Fox | 220 − age |  |
|  |  |  |  |  | Londeree | 206.3 − 0.711 × age |  |
|  |  |  |  |  | Fairbarn | 201 − 0.63 × age |  |
|  |  |  |  |  | Arena | 209.3 − 0.72 × age |  |
| Machado et al. | 2011 | Healthy (male children & adolescents) | 69 | 12.6 ± 1.5 (10 - 16) | Fox | 220 - age | The Fox equation overestimated the measured HR_max_ during a maximal treadmill test and was not valid for this population. The Tanaka equation was valid for this population, showing results that were quite similar to those of measured HR_max_. |
|  |  |  |  |  | Tanaka | 208 - 0.7 × age |  |
| Milani et al. | 2022 | (male & female adults, heart failure patients with reduced ejection fraction) | 191 | 55.8 ± 13.7 | Magri | 109 - (0.5 x age) + (0.5 x resting HR) + (0.2 x LVEF) - (5 if haemoglobin < 11 g/dL)] | Bland–Altman plots indicate a poor agreement between the measured and estimated MHR for both formulas. The Magri equation could still provide the best alternative to predict MHR in HF patients, what can undoubtedly be the best approach prior to a maximum exercise test. |
|  |  |  |  |  | Keteyian | 119 + 0.5 × resting HR - 0.5 × age − (0, for treadmill OR 5, for stationary bike) |  |
| Nikolaidis et al. | 2014 | Athletes (male children & adolescents, team sports) | 147 | 13.6 ± 2.5 (9 - 18) | Fox | 220 - age | Fox and Nikolaidis overestimated measured HR_max_ during an exercise field test (20 m shuttle run test), while Tanaka underestimated it. However, this trend was not consistent when examining each group separately; measured HR_max_ was similar with Tanaka in U-12 and U-15, while it was similar with Nikolaidis in U-18. Tanaka should be preferred when desiring to avoid overtraining, while Fox and Nikolaidis should be the choice in order to ensure adequate exercise intensity. |
|  |  |  |  |  | Tanaka | 208 - 0.7 × age |  |
|  |  |  |  |  | Nikolaidis | 223 − 1.44 × age |  |
| Nikolaidis et al. | 2014 | Athletes (female children & adolescents, volleyball) | 47 | 13.39 ± 2.01 | Fox | 220 - age | Fox overestimated the measured HR_max_ (+5.7 bpm) during an exercise field test (20m shuttle run test), whereas Tanaka was similar to the measured HR_max_ (-2.2 bpm). The Tanaka equation appears to offer a more accurate prediction equation of HR_max_ than the Fox equation in young female volleyball players. |
|  |  |  |  |  | Tanaka | 208 - 0.7 × age |  |
| Papadopoulou et al. | 2019 | Athletes (female adolescents, volleyball players) | 71 | 13.3 ± 0.7 | Fox | 220 - age | Fox overestimated the measured HR_max_ during an exercise field test (20m shuttle run test) by +6.8 bpm and Tanaka underestimated the actual HR_max_ by -2.6 bpm. Age-based prediction equations of HR_max_ developed in adult populations should be applied with caution in physically active female adolescents, and Tanaka should be preferred over the Fox equation. |
|  |  |  |  |  | Tanaka | 208 - 0.7 × age |  |
| Póvoas et al. | 2020 | Athletes (male adults, recreational football players) | 62 | 39.3 ± 5.8 (20 - 50) | Fox | 220 - age | In the untrained status, only the Tanaka (211 - 0.64 × age) and Warburton equations showed non-significant (medium-to-small) differences with measured HR_max_. The Warburton et al. equation can be a practical strategy to limit estimation bias. For the trained status, popular equations like Fox and Tanaka provided HR_max_ estimates that were lower than the SWC. |
|  |  |  |  |  | Fox | 215.4 − 0.9147 × age |  |
|  |  |  |  |  | Londeree | 206.3 - 0.711 × age |  |
|  |  |  |  |  | Inbar | 205.8 - 0.685 × age |  |
|  |  |  |  |  | Tanaka | 208 - 0.7 × age |  |
|  |  |  |  |  | Tanaka | 211 − 0.8 × age |  |
|  |  |  |  |  | Robergs | 208.754 − 0.734 × age |  |
|  |  |  |  |  | Warburton | 226 - age |  |
|  |  |  |  |  | Gellish | 207 - 0.7 × age |  |
|  |  |  |  |  | Nes | 211 - 0.64 × age |  |
| Sarzynski et al. | 2013 | Healthy (male & female adults) | 762 | 16 - 65 | Fox | 220 - age | The standard error of estimate (SEE) of predicted HR_max_ was +12.4 bpm and +11.4 bpm for the Fox and Tanaka formulas, respectively. Based on the SEE, the age-based estimated HR_max_ equations do not precisely predict an individual’s measured HR_max_ measured during maximal exercise tests using cycle ergometers. |
|  |  |  |  |  | Tanaka | 208 - 0.7 × age |  |
| Silva et al. | 2007 | Healthy (female older adults, brazilian) | 93 | 67.12 ± 5.16 | Fox | 220 - age | Fox and Tanaka equations significantly overestimated HR_max_ measured during maximal treadmill tests by a mean difference of 7.4 bpm and 15.5 bpm, respectively. |
|  |  |  |  |  | Tanaka | 208 - 0.7 × age |  |
| Sporis et al. | 2011 | Athletes (military personnel) | 509 | 29.1 ± 5.5 | Fox | 220 - age | The HR_max_ values from Stevens Creek and Fox equations had the highest correlation with the HR_max_ measured during the graded maximal treadmill test. The authors recommend using the HR_max_ values from the Stevens Creek and the Fox equations for the purpose of training, testing, and daily exercise routine in military personnel. |
|  |  |  |  |  | Tanaka | 208 - 0.7 × age |  |
|  |  |  |  |  | Londeree | 206.3 - 0.711 × age |  |
|  |  |  |  |  | Miller | 217 - 0.85 × age |  |
|  |  |  |  |  | Gellish | 206.9 - 0.67 × age |  |
|  |  |  |  |  | Whyte | 202 - 0.55 × age |  |
|  |  |  |  |  | Stevens Creek | 205 - age/2 |  |

HR, heart rate**Table S4a** Grey literature search of user manuals and promotional materials of leading manufacturers.

| **Manufacturer & Model** | | **Prediction of HR_max_** | **Method for prediction of HR_max_** | **Estimation of resting/nocturnal HR** | **Method for measuring/estimating resting/nocturnal HR** | **Link for more info** |
| --- | --- | --- | --- | --- | --- | --- |
| **Amazfit** | Stratos ^a^ | Yes | Not reported | Yes | ‘Resting HR’ can be viewed only after you enable continuous heart rate and wear the watch during sleep more than 5 hours | <https://www.bhphotovideo.com/lit_files/574154.pdf> |
|  | GTR 2 ^b^ | Not reported | Not reported | Not reported | Not disclosed | <https://amazfit-support.cdn.bcebos.com/uploads/doc/20210204/161240828369.pdf> |
|  | T-rex ^c^ | Not reported | Not reported | Yes | Not disclosed | <https://amazfit-support.cdn.bcebos.com/uploads/doc/20220610/165482754148.pdf> |
| **Apple** | Watch Series 8 ^a^ | Yes | Not reported | Yes | Not disclosed | <https://support.apple.com/en-gb/guide/watch/welcome/watchos> |
|  | Watch Nike ^b^ | Yes | Not reported | Yes | Not disclosed | <https://support.apple.com/en-gb/guide/watch/welcome/watchos> |
|  | Watch SE ^c^ | Yes | Not reported | Yes | Not disclosed | <https://support.apple.com/en-gb/guide/watch/welcome/watchos> |
| **Fitbit** | sense 2 a | Yes | 220 - age | Yes | Not disclosed | <https://help.fitbit.com/manuals/sense_2/Content/manuals/html/Get%20Started.htm> |
|  | versa 4 ^b^ | Yes | 220 - age | Yes | Not disclosed | <https://help.fitbit.com/manuals/versa_4/Content/manuals/html/Get%20Started.htm> |
|  | versa 3 ^c^ | Yes | 220 - age | Yes | Not disclosed | <https://help.fitbit.com/manuals/versa_3/Content/manuals/html/Get%20Started.htm> |
| **Garmin** | Forerunner 945 ^a^ | Yes | 220 - age | Yes | Average resting heart rate measured by the watch (Advice: Wear all day the device for the best prediction…) | <https://www8.garmin.com/manuals/webhelp/forerunner945/EN-US/Forerunner_945M_OM_EN-US.pdf> |
|  | Venu 2 series ^b^ | Yes | 220 - age | Yes | Average resting heart rate measured by the watch (Advice: Wear all day the device for the best prediction…) | <https://www8.garmin.com/manuals/webhelp/GUID-D93137A9-B374-4A24-8A4D-A66C9AC91265/EN-US/Venu_2_2S_OM_EN-US.pdf> |
|  | Fénix 7s ^c^ | Yes | 220 - age | Yes | Average resting heart rate measured by the watch (Advice: Wear all day the device for the best prediction…) | <https://www8.garmin.com/manuals/webhelp/GUID-C001C335-A8EC-4A41-AB0E-BAC434259F92/EN-US/fenix_7_Series_OM_EN-US.pdf> |
| **Huawei** | Watch 3 Pro ^a^ | Yes | 220 - age | Yes | Not disclosed | <https://consumer.huawei.com/en/support/content/knowledgelist/en-us-vol15820188/> |
|  | Watch GT Runner ^b^ | Yes | 220 - age | Yes | Not disclosed | <https://consumer.huawei.com/my/support/wearables/watch-gt-runner/> |
|  | Watch Fit 2 ^c^ | Yes | 220 - age | Yes | Not disclosed | <https://consumer.huawei.com/my/support/wearables/watch-fit2/> |
| **Polar** | Pro ^a^ | Yes | 220 - age | Yes | To measure your resting heart rate: 1. Wear your watch. Lie down on your back and relax. 2. After about 1 minute, start a training session on your wearable. Choose any sport profile, for example Other indoor. 3. Lie still and breathe calmly for 3–5 minutes. Don’t look at your training data during the measuring. | <https://support.polar.com/e_manuals/pacer-pro/polar-pacer-pro-user-manual-english/manual.pdf> |
|  | Vantage V2 ^b^ | Yes | 220 - age | Yes | To measure your resting heart rate: 1. Wear your watch. Lie down on your back and relax. 2. After about 1 minute, start a training session on your wearable. Choose any sport profile, for example Other indoor. 3. Lie still and breathe calmly for 3–5 minute | <https://support.polar.com/e_manuals/vantage-v2/polar-vantage-v2-user-manual-english/manual.pdf#page=127&zoom=100,42,644> |
|  | Ignite ^c^ | Yes | 220 - age | Yes | To measure your resting heart rate: 1. Wear your watch. Lie down on your back and relax. 2. After about 1 minute, start a training session on your wearable. Choose any sport profile, for example Other indoor. 3. Lie still and breathe calmly for 3–5 minute | <https://support.polar.com/e_manuals/ignite/polar-ignite-user-manual-english/manual.pdf> |
| **Samsung** | Galaxy Watch5 Pro ^a^ | Yes | 220 - age | Yes | Not disclosed | <https://www.sammobile.com/wp-content/uploads/2022/08/Samsung-Galaxy-Watch-5-Pro-User-Manual.pdf> |
|  | Galaxy Watch 5 ^b^ | Yes | 220 - age | Yes | Not disclosed | <https://www.samsung.com/us/business/support/owners/product/galaxy-watch5-lte/> |
|  | Galaxy Watch 4 ^c^ | Yes | 220 - age | Yes | Not disclosed | <https://www.manua.ls/samsung/galaxy-watch-4/manual?p=3> |
| **Suunto** | Alpha Traverse ^a^ | Not reported | Not reported | Not reported | Not disclosed | <https://ns.suunto.com/Manuals/Traverse_Alpha/Userguides/Suunto_TraverseAlpha_UserGuide_EN.pdf?_ga=2.138893435.951847677.1678092544-355703437.1678092544&_gl=1*n48fsg*_ga*MzU1NzAzNDM3LjE2NzgwOTI1NDQ.*_ga_SGMH8TJLH4*MTY3ODA5MjU0NC4xLjEuMTY3ODA5MzkyMy42MC4wLjA.> |
|  | Spartan Sport Wrist HR ^b^ | Yes | 220 - age | Not reported | Not disclosed | <https://ns.suunto.com/Manuals/Spartan_Sport_WristHR/Userguides/Suunto_Spartan_Sport_WristHR_UserGuide_EN.pdf?_ga=2.93936853.951847677.1678092544-355703437.1678092544&_gl=1*16itazo*_ga*MzU1NzAzNDM3LjE2NzgwOTI1NDQ.*_ga_SGMH8TJLH4*MTY3ODA5MjU0NC4xLjEuMTY3ODA5MzQyNi4xNS4wLjA.> |
|  | 9 Peak Pro ^c^ | Yes | 220 - age | Not reported | Not disclosed | [https://ns.suunto.com/Manuals/Suunto_9_Peak_Pro/Userguides//Suunto_9_Peak_Pro_UserGuide_EN.pdf?_ga=2.172562539.951847677.1678092544-355703437.1678092544&_gl=1*sp5bg4*_ga*MzU1NzAzNDM3LjE2NzgwOTI1NDQ.*_ga_SGMH8TJLH4*MTY3ODA5MjU0NC4xLjEuMTY3ODA5MjcwNi42MC4wLjA.](https://ns.suunto.com/Manuals/Suunto_9_Peak_Pro/Userguides/Suunto_9_Peak_Pro_UserGuide_EN.pdf?_ga=2.172562539.951847677.1678092544-355703437.1678092544&_gl=1*sp5bg4*_ga*MzU1NzAzNDM3LjE2NzgwOTI1NDQ.*_ga_SGMH8TJLH4*MTY3ODA5MjU0NC4xLjEuMTY3ODA5MjcwNi42MC4wLjA.) |
| **Xiaomi** | Mi watch ^a^ | Not reported | Not reported | Not reported | Not disclosed | No user manual available |
|  | Watch S1 Active ^b^ | Not reported | Not reported | Not reported | Not disclosed | No user manual available |
|  | Haylou Ls05 ^c^ | Not reported | Not reported | Not reported | Not disclosed | No user manual available |

^a^, ^b^ and ^c^ refer to a high-, medium- and low-grade device, respectively; HR, heart rate.

**Table S4b** Grey literature search of user manuals and promotional materials of mobile applications associated with smart watches of leading manufacturers.

| **App to display activity zones** | **How continuous HR data is used to estimate activity zones in the app** | **Export of 24hr continuous HR data** | **Link for more info** |
| --- | --- | --- | --- |
| **Zepp (Amazfit & Xiaomi)** | 6 Zones based and HR_max_ (220 - age).  Relaxed (<50%) Light (50% - 60%) Intensive (60% - 70%) Aerobic (80% - 90%) Anaerobic (90% - 99%) VO_2_Max (maximal HR)  Additionally absoulte and realtive time spent in each zone is displayed. | Yes - Data can be exported and send to user mail directly in the app (Profile > Settings > Information > User Rights > Export Data) | <https://support.amazfit.com/en/faq/797> |
|  |  |  |  |
|  |  |  |  |
| **watchOS 9 (Apple)** | Continuous HR is displayed as HR ranges during certian time intervals (e.g. minimal and maximal HR during in a hour or during the day) in form of a bar graph. HR zones are not specified.  Daily actvity is diplayed as distance, standing minutes, and exercise minutes. | Yes - can be exported directly from the app in .xml or .csv format or via third-party apps that are more user friendly. | [https://support.apple.com/guide/watch/apd3bf6d85a6/watchos  https://support.apple.com/en-us/HT204666#:~:text=When%20Apple%20Watch%20measures%20your,heart%20rate%2C%20check%20your%20settings.  https://discussions.apple.com/thread/8254597#:~:text=On%20your%20iPhone%2C%20in%20the,a%20more%20user%2Dfriendly%20format.](https://support.apple.com/guide/watch/apd3bf6d85a6/watchos) |
|  |  |  |  |
|  |  |  |  |
| **Fitbit app** | HR zones are calculated based on heart rate reserve (HRmax (220 -age) - resting HR).   Fat burn zone (40% to 59% of HRR) Cardio Zone (60% to 84% of HRR)  Peak Zone (≥85% of HRR)  Additionally Active Zone Minutes are calculated for time spent in the fat burn, cardio, or peak heart-rate zones based on HR. 2x Active Zone Minutes for time spent in your cardio or peak heart rate zone. | Yes - Data can be exported as .xlsx or .csv file. | [https://help.fitbit.com/articles/en_US/Help_article/1565.htm  https://help.fitbit.com/articles/en_US/Help_article/1379.htm  https://blog.fitbit.com/active-zone-minutes/#:~:text=The%20three%20target%20heart%20rate,biking%2C%20or%20a%20HIIT%20workout.](https://help.fitbit.com/articles/en_US/Help_article/1379.htm) |
|  |  |  |  |
|  |  |  |  |
| **Garmin connect** | HR data and accelerometer data are displayed in 6 intensity leves:  Zone <1 (Everything below training zones) Zone 1 (Warm Up) Zone 2 (Easy) Zone 3 (Aerobic) Zone 4 (Threshold) Zone 5 (Maximum)  graphically displayed over 24hrs and as intensity minutes (time spent with moderate and vigurous activity per day) | Yes - Data can be downloaded as .fit files. The GARMIN FIT SDK developer tool (https://developer.garmin.com/fit/overview/) can be used to convert data to .csv file for further data analysis | <https://support.garmin.com/en-US/?faq=s3HqdKNtWV1NYrK16eFcc7> |
|  |  |  |  |
|  |  |  |  |
| **HUAWEI HEALTH** | Daily continuous HR data is just displayed over 24 hours. No seperation into zones. Daily activity is mainly displayed as steps and activity times based on accelerometer data. | Yes - But only via Health Kit SDK developer tool (https://developer.huawei.com/consumer/en/doc/HMS-Plugin-Guides/read-sport-health-datatype-0000001073827835-V1) | <https://consumer.huawei.com/mobileservices/health/> |
|  |  |  |  |
|  |  |  |  |
| **Polar flow** | HR data and accelerometer data are displayed in 5 intensity leves:  1. resting (sleeping and resting while lying down) 2. sitting (sitting and other passive behaviour) 3. low (standing work, light housework) 4. medium (walking and other moderate activities) 5. high (jogging, running and other intense activities)  graphically displayed over 24hrs and as time spent in each zone per day | No - Only export of HR data from training sessions possible | [https://support.polar.com/en/support/the_what_and_how_of_polars_continuous_heart_rate & https://support.polar.com/en/support/the_what_and_how_of_polar_24_7_activity_tracking](https://support.polar.com/en/support/the_what_and_how_of_polars_continuous_heart_rate&) |
|  |  |  |  |
|  |  |  |  |
| **SAMSUNG HEALTH** | Continuous HR is displayed as HR ranges during certian time intervals (e.g. minimal and maximal HR during in a hour or during the day) in form of a bar graph. HR zones are not specified.  Just daily steps and active time based on acceleromter data is displayed to display daily activity. | Yes - can be exported directly from the app in .csv or .json format. | <https://www.samsung.com/support/apps-services/how-samsung-health-monitors-your-heart-rate/> |
|  |  |  |  |
|  |  |  |  |
| **Suunto app** | Daily continuous HR data is just displayed over 24 hours. No seperation into zones. Daily activity is mainly displayed as steps. | No - Only export of HR data from training sessions possible | <https://www.suunto.com/de-de/Support/faq-articles/suunto-app/welche-dateiformate-kann-ich-aus-der-suunto-app-exportieren/> |
